# Supplementary figures and images for: Genome-wide association study of cassava starch paste properties
Source: PLoS One. 2022 Jan 21;17(1):e0262888. doi: 10.1371/journal.pone.0262888 (PMC8782291; doi:10.1371/journal.pone.0262888)

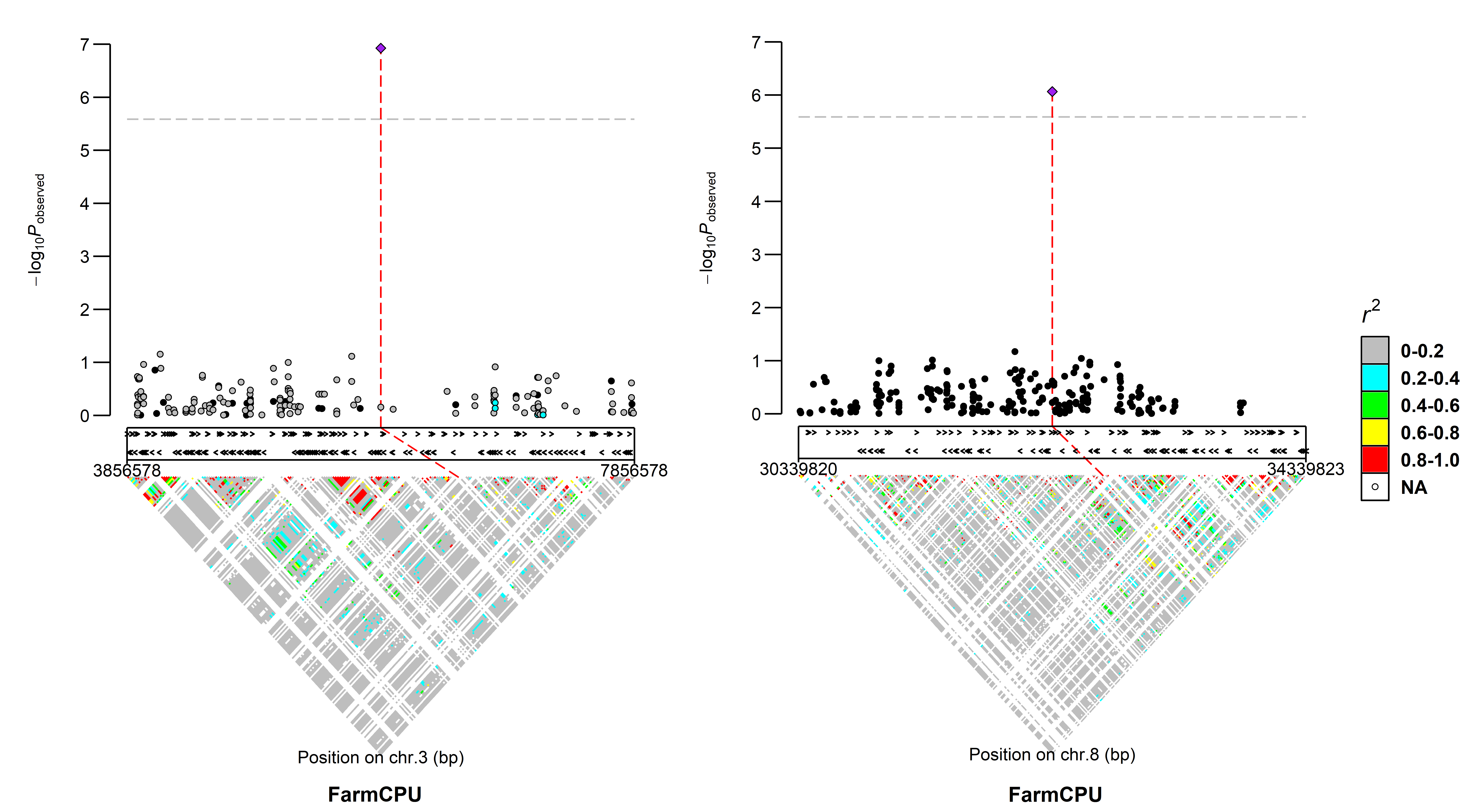

Supplement: S2 Fig — The middle layer shows the filtered genes and annotated sequences of the cassava genome (Phytozome v12.1) and the bottom layer shows the linkage disequilibrium matrix. (TIF) [file pone.0262888.s002.tif]

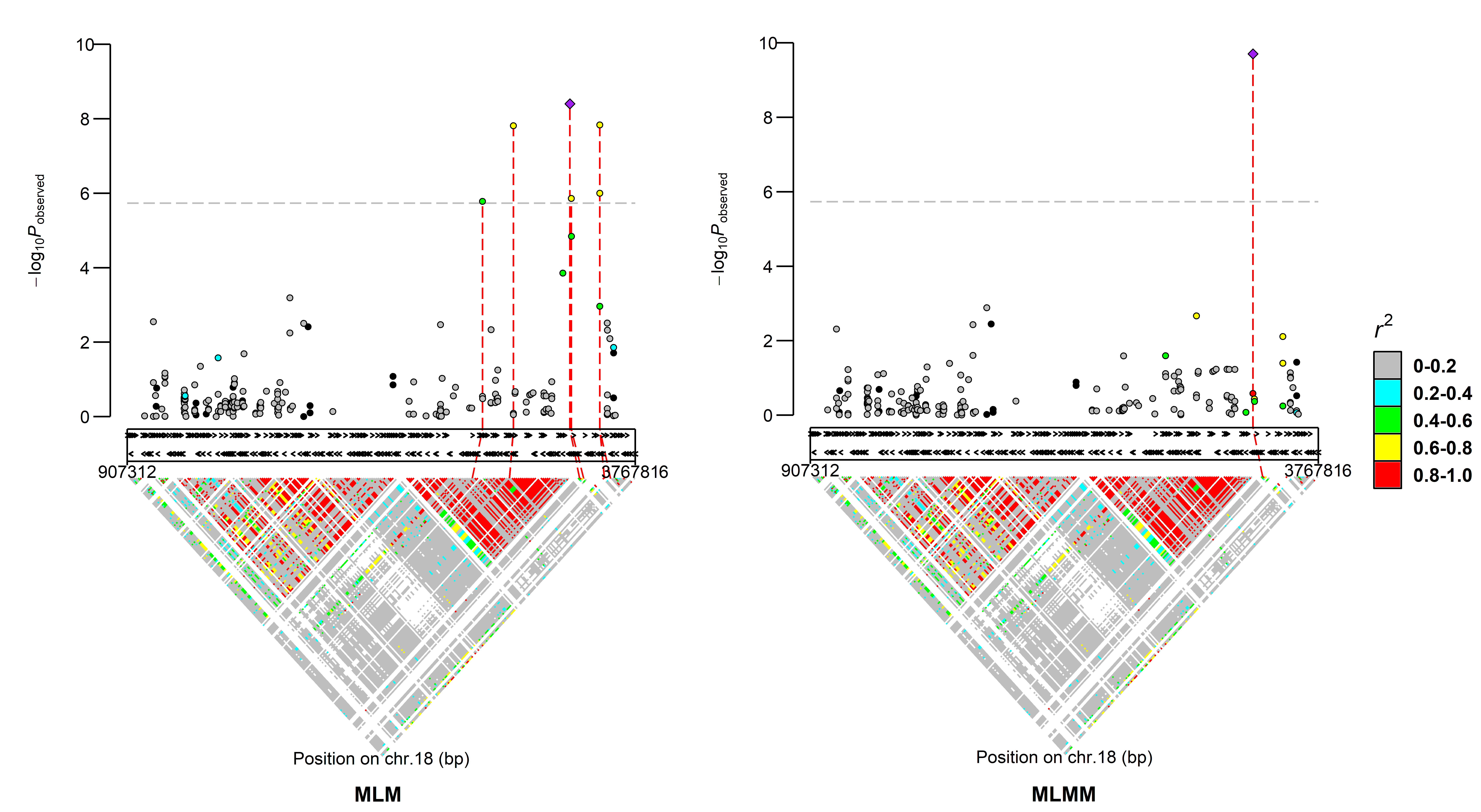

Supplement: S6 Fig — The middle layer shows the filtered genes and annotated sequences of the cassava genome (Phytozome v12.1) and the bottom layer shows the linkage disequilibrium matrix. (TIF) [file pone.0262888.s006.tif]
