# Supplementary material for: Genome-wide association study of cassava starch paste properties
Source: PLoS One. 2022 Jan 21;17(1):e0262888. doi: 10.1371/journal.pone.0262888 (PMC8782291; doi:10.1371/journal.pone.0262888)
Supplement: S1 Table — (DOCX) [file pone.0262888.s007.docx]

**Genome-wide association study of cassava starch paste properties**

**S1 Table**. List of cassava accessions with geographical and genetic origin, state of collection, and entry date on the Embrapa’s cassava germplasm bank.

| **Clone1** | **PCA cluster** | **Geographical origin** | **State** | **Genetic origin** | **Entry date** |
| --- | --- | --- | --- | --- | --- |
| BGM-0540 | 1 | North East Brazil | Alagoas | Landrace | 1978 |
| BGM-0984 | 1 | North Brazil | Amazonas | Landrace | 1987 |
| BRS Novo Horizonte | 1 | North East Brazil | Bahia | Improved variety | 1998 |
| BGM-1657 | 1 | North East Brazil | Bahia | Improved variety | 1996 |
| BGM-0726 | 1 | North East Brazil | Bahia | Improved variety | 1981 |
| BGM-0111 | 1 | North East Brazil | Bahia | Landrace | 1971 |
| BGM-0140 | 1 | North East Brazil | Bahia | Landrace | 1971 |
| BGM-1024 | 1 | North East Brazil | Bahia | Landrace | 1987 |
| BGM-1496 | 1 | North East Brazil | Bahia | Landrace | 1994 |
| BGM-1547 | 1 | North East Brazil | Bahia | Landrace | 1994 |
| BGM-1088 | 1 | North East Brazil | Bahia | Landrace | 1987 |
| BGM-1118 | 1 | North East Brazil | Bahia | Landrace | 1987 |
| BGM-0134 | 1 | North East Brazil | Bahia | Landrace | 1971 |
| BGM-1457 | 1 | North East Brazil | Bahia | Landrace | 1992 |
| BGM-1473 | 1 | North East Brazil | Bahia | Landrace | 1994 |
| BGM-1475 | 1 | North East Brazil | Bahia | Landrace | 1994 |
| BGM-1477 | 1 | North East Brazil | Bahia | Landrace | 1994 |
| BGM-1494 | 1 | North East Brazil | Bahia | Landrace | 1994 |
| BGM-1497 | 1 | North East Brazil | Bahia | Landrace | 1994 |
| BGM-1502 | 1 | North East Brazil | Bahia | Landrace | 1994 |
| BGM-0153 | 1 | North East Brazil | Bahia | Landrace | 1972 |
| BGM-1537 | 1 | North East Brazil | Bahia | Landrace | 1994 |
| BGM-1549 | 1 | North East Brazil | Bahia | Landrace | 1994 |
| BGM-0161 | 1 | North East Brazil | Bahia | Landrace | 1972 |
| BGM-1660 | 1 | North East Brazil | Bahia | Landrace | 1996 |
| BGM-0222 | 1 | North East Brazil | Bahia | Landrace | 1972 |
| BGM-0226 | 1 | North East Brazil | Bahia | Landrace | 1972 |
| BGM-0255 | 1 | North East Brazil | Bahia | Landrace | 1971 |
| BGM-0497 | 1 | North East Brazil | Bahia | Landrace | 1978 |
| BGM-0050 | 1 | North East Brazil | Bahia | Landrace | 1966 |
| BGM-0064 | 1 | North East Brazil | Bahia | Landrace | 1972 |
| BGM-0074 | 1 | North East Brazil | Bahia | Landrace | 1972 |
| BGM-0094 | 1 | North East Brazil | Bahia | Landrace | 1972 |
| BGM-0606 | 1 | North East Brazil | Ceará | Landrace | 1980 |
| BGM-0610 | 1 | North East Brazil | Ceará | Landrace | 1980 |
| BGM-0624 | 1 | North East Brazil | Ceará | Landrace | 1980 |
| BGM-0656 | 1 | North East Brazil | Ceará | Landrace | 1981 |
| BGM-0929 | 1 | Midwest Brazil | Distrito Federal | Improved variety | 1987 |
| BGM-0496 | 1 | Southeast Brazil | Espírito Santo | Landrace | 1977 |
| BGM-1772 | 1 | North East Brazil | Maranhão | Landrace | 2001 |
| BGM-1102 | 1 | Midwest Brazil | Mato Grosso | Landrace | 1987 |
| BGM-1456 | 1 | Midwest Brazil | Mato Grosso | Landrace | 1990 |
| BGM-0507 | 1 | Midwest Brazil | Mato Grosso | Landrace | 1977 |
| BGM-1698 | 1 | Midwest Brazil | Mato Grosso do Sul | Landrace | 2001 |
| BGM-1699 | 1 | Midwest Brazil | Mato Grosso do Sul | Landrace | 2001 |
| BGM-0584 | 1 | Southeast Brazil | Minas Gerais | Landrace | 1978 |
| BGM-0413 | 1 | Southeast Brazil | Minas Gerais | Landrace | 1979 |
| BGM-0043 | 1 | Southeast Brazil | Minas Gerais | Landrace | 1968 |
| BGM-0464 | 1 | Southeast Brazil | Minas Gerais | Landrace | 1977 |
| BGM-0509 | 1 | Southeast Brazil | Minas Gerais | Landrace | 1977 |
| BGM-0006 | 1 | Southeast Brazil | Minas Gerais | Landrace | 1968 |
| BGM-0908 | 1 | Venezuela | NA | Landrace | 1976 |
| BGM-1251 | 1 | Nigeria | NA | Improved variety | 1989 |
| BGM-0423 | 1 | Brazil-NA | NA | Improved variety | 1977 |
| BGM-0514 | 1 | Brazil-NA | NA | Improved variety | 1977 |
| BGM-0533 | 1 | Brazil-NA | NA | Improved variety | 1976 |
| BGM-0017 | 1 | Brazil-NA | NA | Landrace | 1967 |
| BGM-1218 | 1 | Brazil-NA | NA | Landrace | 1989 |
| BGM-1222 | 1 | Brazil-NA | NA | Landrace | 1989 |
| BGM-0362 | 1 | Brazil-NA | NA | Landrace | 1972 |
| BGM-0409 | 1 | Brazil-NA | NA | Landrace | 1977 |
| BGM-0437 | 1 | Brazil-NA | NA | Landrace | 1977 |
| BGM-0529 | 1 | Brazil-NA | NA | Landrace | 1977 |
| BGM-0057 | 1 | Brazil-NA | NA | Landrace | 1973 |
| BGM-1160 | 1 | North Brazil | Pará | Landrace | 1988 |
| BGM-1637 | 1 | North Brazil | Pará | Landrace | 1995 |
| BGM-0467 | 1 | North Brazil | Pará | Landrace | 1977 |
| BGM-1373 | 1 | North East Brazil | Paraíba | Landrace | 1991 |
| BGM-1082 | 1 | North East Brazil | Paraíba | Landrace | 1987 |
| BGM-1360 | 1 | North East Brazil | Paraíba | Landrace | 1991 |
| BGM-1364 | 1 | North East Brazil | Paraíba | Landrace | 1991 |
| BGM-1367 | 1 | North East Brazil | Paraíba | Landrace | 1991 |
| BGM-1622 | 1 | North East Brazil | Paraíba | Landrace | 1995 |
| Fécula Branca | 1 | South Brazil | Paraná | Improved variety | 2008 |
| IAC90 | 1 | South Brazil | Paraná | Improved Variety | 2012 |
| AmarelaSC | 1 | South Brazil | Paraná | Landrace | 2012 |
| BGM-1311 | 1 | North East Brazil | Pernambuco | Landrace | 1991 |
| BGM-1316 | 1 | North East Brazil | Pernambuco | Landrace | 1991 |
| BGM-1335 | 1 | North East Brazil | Pernambuco | Landrace | 1991 |
| BGM-1269 | 1 | North East Brazil | Pernambuco | Landrace | 1991 |
| BGM-1270 | 1 | North East Brazil | Pernambuco | Landrace | 1991 |
| BGM-1274 | 1 | North East Brazil | Pernambuco | Landrace | 1991 |
| BGM-1275 | 1 | North East Brazil | Pernambuco | Landrace | 1991 |
| BGM-1285 | 1 | North East Brazil | Pernambuco | Landrace | 1991 |
| BGM-1628 | 1 | North East Brazil | Pernambuco | Landrace | 1995 |
| BGM-0570 | 1 | North East Brazil | Pernambuco | Landrace | 1978 |
| BGM-1566 | 1 | North East Brazil | Piauí | Landrace | 1994 |
| BGM-1583 | 1 | North East Brazil | Piauí | Landrace | 1994 |
| BGM-1602 | 1 | North East Brazil | Piauí | Landrace | 1994 |
| BGM-0461 | 1 | Southeast Brazil | Rio de Janeiro | Improved variety | 1977 |
| BGM-0494 | 1 | Southeast Brazil | Rio de Janeiro | Landrace | 1977 |
| BGM-0495 | 1 | Southeast Brazil | Rio de Janeiro | Landrace | 1977 |
| BGM-1067 | 1 | North East Brazil | Rio Grande do Norte | Landrace | 1987 |
| BGM-1398 | 1 | North East Brazil | Rio Grande do Norte | Landrace | 1991 |
| BGM-1130 | 1 | North East Brazil | Rio Grande do Norte | Landrace | 1987 |
| BGM-1422 | 1 | North East Brazil | Rio Grande do Norte | Landrace | 1991 |
| BGM-2071 | 1 | South Brazil | Rio Grande do Sul | Improved variety | 2011 |
| BGM-0095 | 1 | South Brazil | Rio Grande do Sul | Landrace | 1972 |
| BGM-0593 | 1 | South Brazil | Rio Grande do Sul | Landrace | 1978 |
| BGM-0598 | 1 | South Brazil | Rio Grande do Sul | Landrace | 1978 |
| BGM-1131 | 1 | Southeast Brazil | São Paulo | Improved variety | 1983 |
| BGM-0020 | 1 | Southeast Brazil | São Paulo | Improved variety | 1967 |
| BGM-0352 | 1 | Southeast Brazil | São Paulo | Improved variety | 1977 |
| BGM-0357 | 1 | Southeast Brazil | São Paulo | Improved variety | 1977 |
| BGM-0407 | 1 | Southeast Brazil | São Paulo | Improved variety | 1977 |
| BGM-0421 | 1 | Southeast Brazil | São Paulo | Improved variety | 1977 |
| BGM-0440 | 1 | Southeast Brazil | São Paulo | Improved variety | 1977 |
| BGM-1633 | 1 | North East Brazil | Sergipe | Landrace | 1995 |
| BGM-0056 | 1 | North East Brazil | Sergipe | Landrace | 1972 |
| BGM-1119 | 1 | North Brazil | Tocantins | Landrace | 1987 |
| BGM-0340 | 1 | Colombia | Valle | Improved Variety | 1976 |
| BGM-0290 | 1 | Colombia | ValledelCauca | Improved variety | 1976 |
| BGM-0315 | 1 | Colombia | ValledelCauca | Improved variety | 1976 |
| BGM-1236 | 1 | Colombia | ValledelCauca | Improved variety | 1989 |
| BGM-1243 | 1 | Colombia | ValledelCauca | Improved variety | 1989 |
| BGM-1183 | 2 | North Brazil | Acre | Landrace | 1989 |
| BGM-0706 | 2 | North Brazil | Acre | Landrace | 1981 |
| BGM-1198 | 2 | North Brazil | Amapá | Landrace | 1989 |
| BGM-1700 | 2 | North Brazil | Amazonas | Improved variety | 2001 |
| BGM-1703 | 2 | North Brazil | Amazonas | Improved variety | 2001 |
| BGM-1704 | 2 | North Brazil | Amazonas | Improved variety | 2001 |
| BGM-1705 | 2 | North Brazil | Amazonas | Improved variety | 2001 |
| BGM-1710 | 2 | North Brazil | Amazonas | Improved variety | 2001 |
| BGM-1711 | 2 | North Brazil | Amazonas | Improved variety | 2001 |
| BGM-0943 | 2 | North Brazil | Amazonas | Landrace | 1987 |
| BGM-0944 | 2 | North Brazil | Amazonas | Landrace | 1987 |
| BGM-0952 | 2 | North Brazil | Amazonas | Landrace | 1987 |
| BGM-0953 | 2 | North Brazil | Amazonas | Landrace | 1987 |
| BGM-0958 | 2 | North Brazil | Amazonas | Landrace | 1987 |
| BGM-0960 | 2 | North Brazil | Amazonas | Landrace | 1987 |
| BGM-0961 | 2 | North Brazil | Amazonas | Landrace | 1987 |
| BGM-0962 | 2 | North Brazil | Amazonas | Landrace | 1987 |
| BGM-0965 | 2 | North Brazil | Amazonas | Landrace | 1987 |
| BGM-0972 | 2 | North Brazil | Amazonas | Landrace | 1987 |
| BGM-0973 | 2 | North Brazil | Amazonas | Landrace | 1987 |
| BGM-0975 | 2 | North Brazil | Amazonas | Landrace | 1987 |
| BGM-0981 | 2 | North Brazil | Amazonas | Landrace | 1987 |
| BGM-0982 | 2 | North Brazil | Amazonas | Landrace | 1987 |
| BGM-0983 | 2 | North Brazil | Amazonas | Landrace | 1987 |
| BGM-0987 | 2 | North Brazil | Amazonas | Landrace | 1987 |
| BGM-0989 | 2 | North Brazil | Amazonas | Landrace | 1987 |
| BGM-1132 | 2 | North Brazil | Amazonas | Landrace | 1988 |
| BGM-1133 | 2 | North Brazil | Amazonas | Landrace | 1988 |
| BGM-1135 | 2 | North Brazil | Amazonas | Landrace | 1988 |
| BGM-1137 | 2 | North Brazil | Amazonas | Landrace | 1988 |
| BGM-1138 | 2 | North Brazil | Amazonas | Landrace | 1988 |
| BGM-1139 | 2 | North Brazil | Amazonas | Landrace | 1988 |
| BGM-1141 | 2 | North Brazil | Amazonas | Landrace | 1988 |
| BGM-1142 | 2 | North Brazil | Amazonas | Landrace | 1988 |
| BGM-1144 | 2 | North Brazil | Amazonas | Landrace | 1988 |
| BGM-1185 | 2 | North Brazil | Amazonas | Landrace | 1989 |
| BGM-0132 | 2 | North Brazil | Amazonas | Landrace | 1972 |
| BGM-1884 | 2 | North Brazil | Amazonas | Landrace | 2006 |
| BGM-1957 | 2 | North Brazil | Amazonas | Landrace | 2007 |
| BGM-1980 | 2 | North Brazil | Amazonas | Landrace | 2006 |
| BGM-0907 | 2 | North Brazil | Amazonas | Landrace | 1987 |
| BGM-0565 | 2 | North East Brazil | Bahia | Improved variety | 1978 |
| BGM-0728 | 2 | North East Brazil | Bahia | Improved variety | 1981 |
| BGM-0729 | 2 | North East Brazil | Bahia | Improved variety | 1981 |
| BRS Formosa | 2 | North East Brazil | Bahia | Improved variety | 2002 |
| BGM-0936 | 2 | North East Brazil | Bahia | Landrace | 1987 |
| BGM-0188 | 2 | North East Brazil | Bahia | Landrace | 1971 |
| BGM-0257 | 2 | North East Brazil | Bahia | Landrace | 1971 |
| BGM-0585 | 2 | North East Brazil | Bahia | Landrace | 1972 |
| BGM-0073 | 2 | North East Brazil | Bahia | Landrace | 1972 |
| BGM-0776 | 2 | North East Brazil | Bahia | Landrace | 1981 |
| BGM-0364 | 2 | North East Brazil | Ceará | Landrace | 1977 |
| BGM-0380 | 2 | North East Brazil | Ceará | Landrace | 1977 |
| BGM-0733 | 2 | Southeast Brazil | Espírito Santo | Landrace | 1981 |
| BGM-0752 | 2 | Southeast Brazil | Espírito Santo | Landrace | 1981 |
| BGM-0699 | 2 | North East Brazil | Maranhão | Landrace | 1981 |
| BGM-1730 | 2 | North East Brazil | Maranhão | Landrace | 2001 |
| BGM-1733 | 2 | North East Brazil | Maranhão | Landrace | 2001 |
| BGM-1734 | 2 | North East Brazil | Maranhão | Landrace | 2001 |
| BGM-1742 | 2 | North East Brazil | Maranhão | Landrace | 2001 |
| BGM-1746 | 2 | North East Brazil | Maranhão | Landrace | 2001 |
| BGM-1747 | 2 | North East Brazil | Maranhão | Landrace | 2001 |
| BGM-1756 | 2 | North East Brazil | Maranhão | Landrace | 2001 |
| BGM-1768 | 2 | North East Brazil | Maranhão | Landrace | 2001 |
| BGM-1769 | 2 | North East Brazil | Maranhão | Landrace | 2001 |
| BGM-1780 | 2 | North East Brazil | Maranhão | Landrace | 2001 |
| BGM-1794 | 2 | North East Brazil | Maranhão | Landrace | 2001 |
| BGM-0660 | 2 | North East Brazil | Maranhão | Landrace | 1981 |
| BGM-0662 | 2 | North East Brazil | Maranhão | Landrace | 1981 |
| BGM-0669 | 2 | North East Brazil | Maranhão | Landrace | 1981 |
| BGM-0673 | 2 | North East Brazil | Maranhão | Landrace | 1981 |
| BGM-0677 | 2 | North East Brazil | Maranhão | Landrace | 1981 |
| BGM-0678 | 2 | North East Brazil | Maranhão | Landrace | 1981 |
| BGM-0679 | 2 | North East Brazil | Maranhão | Landrace | 1981 |
| BGM-0685 | 2 | North East Brazil | Maranhão | Landrace | 1981 |
| BGM-0695 | 2 | North East Brazil | Maranhão | Landrace | 1981 |
| BGM-0700 | 2 | North East Brazil | Maranhão | Landrace | 1981 |
| BGM-1143 | 2 | Midwest Brazil | Mato Grosso | Landrace | 1988 |
| BGM-0517 | 2 | Southeast Brazil | Minas Gerais | Landrace | 1977 |
| BGM-0424 | 2 | Brazil-NA | NA | Landrace | 1977 |
| BGM-0450 | 2 | Brazil-NA | NA | Landrace | 1977 |
| BGM-1158 | 2 | Brazil-NA | NA | Landrace | 1988 |
| BGM-1159 | 2 | Brazil-NA | NA | Landrace | 1988 |
| BGM-1162 | 2 | Brazil-NA | NA | Landrace | 1988 |
| BGM-1164 | 2 | Brazil-NA | NA | Landrace | 1988 |
| BGM-1177 | 2 | Brazil-NA | NA | Landrace | 1988 |
| BGM-1178 | 2 | Brazil-NA | NA | Landrace | 1988 |
| BGM-1184 | 2 | Brazil-NA | NA | Landrace | 1989 |
| BGM-1448 | 2 | Brazil-NA | NA | Landrace | 1991 |
| BGM-0058 | 2 | Brazil-NA | NA | Landrace | 1972 |
| BGM-0914 | 2 | North Brazil | Pará | Landrace | 1987 |
| BGM-0940 | 2 | North Brazil | Pará | Landrace | 1987 |
| BGM-1171 | 2 | North Brazil | Pará | Landrace | 1988 |
| BGM-1190 | 2 | North Brazil | Pará | Landrace | 1989 |
| BGM-1209 | 2 | North Brazil | Pará | Landrace | 1989 |
| BGM-1210 | 2 | North Brazil | Pará | Landrace | 1989 |
| BGM-1214 | 2 | North Brazil | Pará | Landrace | 1989 |
| BGM-1674 | 2 | North Brazil | Pará | Landrace | 1999 |
| BGM-1675 | 2 | North Brazil | Pará | Landrace | 1999 |
| BGM-0019 | 2 | North Brazil | Pará | Landrace | 1967 |
| BGM-1942 | 2 | North Brazil | Pará | Landrace | 2006 |
| BGM-0054 | 2 | North Brazil | Pará | Landrace | 1972 |
| BGM-0061 | 2 | North Brazil | Pará | Landrace | 1972 |
| BGM-0066 | 2 | North Brazil | Pará | Landrace | 1972 |
| BGM-0067 | 2 | North Brazil | Pará | Landrace | 1972 |
| BGM-0070 | 2 | North Brazil | Pará | Landrace | 1972 |
| BGM-0895 | 2 | North Brazil | Pará | Landrace | 1982 |
| BGM-0902 | 2 | North Brazil | Pará | Landrace | 1982 |
| BGM-1179 | 2 | North East Brazil | Paraíba | Landrace | 1988 |
| BGM-1354 | 2 | North East Brazil | Paraíba | Landrace | 1991 |
| BGM-1450 | 2 | South Brazil | Paraná | Landrace | 1991 |
| BGM-1280 | 2 | North East Brazil | Pernambuco | Landrace | 1991 |
| BGM-1284 | 2 | North East Brazil | Pernambuco | Landrace | 1991 |
| BGM-1286 | 2 | North East Brazil | Pernambuco | Landrace | 1991 |
| BGM-2028 | 2 | North East Brazil | Pernambuco | Landrace | 2006 |
| BGM-2029 | 2 | North East Brazil | Pernambuco | Landrace | 2006 |
| BGM-0587 | 2 | North East Brazil | Pernambuco | Landrace | 1978 |
| BGM-1043 | 2 | North East Brazil | Piauí | Landrace | 1987 |
| BGM-1605 | 2 | North East Brazil | Piauí | Landrace | 1994 |
| BGM-0468 | 2 | Southeast Brazil | Rio de Janeiro | Improved variety | 1977 |
| BGM-1415 | 2 | North East Brazil | Rio Grande do Norte | Landrace | 1991 |
| BGM-0597 | 2 | South Brazil | Rio Grande do Sul | Landrace | 1978 |
| BGM-0661 | 2 | South Brazil | Rio Grande do Sul | Landrace | 1981 |
| BGM-0267 | 2 | Southeast Brazil | São Paulo | Improved variety | 1972 |
| BGM-0269 | 2 | North East Brazil | Sergipe | Landrace | 1972 |
| BGM-0819 | 2 | North East Brazil | Sergipe | Landrace | 1981 |
| BGM-1878 | 2 | North Brazil | Tocantins | Landrace | 2005 |
| BGM-0889 | 2 | North Brazil | Tocantins | Landrace | 1982 |
| BGM-0904 | 2 | Colombia | Valle | Improved Variety | 1982 |
| BGM-0996 | 2 | Colombia | ValledelCauca | Improved variety | 1987 |
| BGM-1213 | 2 | Colombia | ValledelCauca | Improved variety | 1989 |
| BGM-0327 | 2 | Colombia | ValledelCauca | Improved variety | 1976 |
| BGM-0342 | 2 | Colombia | ValledelCauca | Improved variety | 1976 |
| BGM-0345 | 2 | Colombia | ValledelCauca | Improved variety | 1976 |
| BGM-0718 | 3 | North Brazil | Acre | Landrace | 1981 |
| BGM-1639 | 3 | North East Brazil | Alagoas | Landrace | 1995 |
| BGM-0545 | 3 | North East Brazil | Alagoas | Landrace | 1978 |
| BGM-0817 | 3 | North East Brazil | Alagoas | Landrace | 1981 |
| BGM-1706 | 3 | North Brazil | Amazonas | Improved variety | 2001 |
| BGM-1716 | 3 | North Brazil | Amazonas | Improved variety | 2001 |
| BGM-0979 | 3 | North Brazil | Amazonas | Landrace | 1987 |
| BGM-0992 | 3 | North Brazil | Amazonas | Landrace | 1987 |
| BGM-0993 | 3 | North Brazil | Amazonas | Landrace | 1987 |
| 7745_5wx | 3 | North East Brazil | Bahia | Improved variety | 2010 |
| BGM-1728 | 3 | North East Brazil | Bahia | Improved variety | 2001 |
| BGM-2041 | 3 | North East Brazil | Bahia | Improved variety | 2009 |
| BGM-0014 | 3 | North East Brazil | Bahia | Improved variety | 1965 |
| BGM-0201 | 3 | North East Brazil | Bahia | Improved Variety | 1974 |
| BRS Jari | 3 | North East Brazil | Bahia | Improved Variety | 2009 |
| BRS Kiriris | 3 | North East Brazil | Bahia | Improved Variety | 2001 |
| BGM-0063 | 3 | North East Brazil | Bahia | Landrace | 1972 |
| BGM-0202 | 3 | North East Brazil | Bahia | Landrace | 1971 |
| BGM-0252 | 3 | North East Brazil | Bahia | Landrace | 1972 |
| BGM-0935 | 3 | North East Brazil | Bahia | Landrace | 1987 |
| BGM-1491 | 3 | North East Brazil | Bahia | Landrace | 1994 |
| BGM-1817 | 3 | North East Brazil | Bahia | Landrace | 2003 |
| BGM-0256 | 3 | North East Brazil | Bahia | Landrace | 1967 |
| BGM-1127 | 3 | North East Brazil | Bahia | Landrace | 1987 |
| BGM-1203 | 3 | North East Brazil | Bahia | Landrace | 1989 |
| BGM-1206 | 3 | North East Brazil | Bahia | Landrace | 1989 |
| BGM-0123 | 3 | North East Brazil | Bahia | Landrace | 1972 |
| BGM-1442 | 3 | North East Brazil | Bahia | Landrace | 1991 |
| BGM-0145 | 3 | North East Brazil | Bahia | Landrace | 1972 |
| BGM-1465 | 3 | North East Brazil | Bahia | Landrace | 1993 |
| BGM-1472 | 3 | North East Brazil | Bahia | Landrace | 1994 |
| BGM-1474 | 3 | North East Brazil | Bahia | Landrace | 1994 |
| BGM-1488 | 3 | North East Brazil | Bahia | Landrace | 1994 |
| BGM-1508 | 3 | North East Brazil | Bahia | Landrace | 1994 |
| BGM-1517 | 3 | North East Brazil | Bahia | Landrace | 1994 |
| BGM-1543 | 3 | North East Brazil | Bahia | Landrace | 1994 |
| BGM-1631 | 3 | North East Brazil | Bahia | Landrace | 1995 |
| BGM-1683 | 3 | North East Brazil | Bahia | Landrace | 1999 |
| BGM-1692 | 3 | North East Brazil | Bahia | Landrace | 1999 |
| BGM-1721 | 3 | North East Brazil | Bahia | Landrace | 2001 |
| BGM-1722 | 3 | North East Brazil | Bahia | Landrace | 2001 |
| BGM-1818 | 3 | North East Brazil | Bahia | Landrace | 2003 |
| BGM-0187 | 3 | North East Brazil | Bahia | Landrace | 1972 |
| BGM-0204 | 3 | North East Brazil | Bahia | Landrace | 1974 |
| BGM-0212 | 3 | North East Brazil | Bahia | Landrace | 1972 |
| BGM-0213 | 3 | North East Brazil | Bahia | Landrace | 1969 |
| BGM-0248 | 3 | North East Brazil | Bahia | Landrace | 1972 |
| BGM-0250 | 3 | North East Brazil | Bahia | Landrace | 1971 |
| BGM-0254 | 3 | North East Brazil | Bahia | Landrace | 1971 |
| BGM-0288 | 3 | North East Brazil | Bahia | Landrace | 1972 |
| BGM-0562 | 3 | North East Brazil | Bahia | Landrace | 1978 |
| BGM-0062 | 3 | North East Brazil | Bahia | Landrace | 1969 |
| BGM-0800 | 3 | North East Brazil | Bahia | Landrace | 1981 |
| BRS Dourada | 3 | North East Brazil | Bahia | Landrace | 1999 |
| BGM-1042 | 3 | North East Brazil | Ceará | Landrace | 1987 |
| BGM-0365 | 3 | North East Brazil | Ceará | Landrace | 1977 |
| BGM-0369 | 3 | North East Brazil | Ceará | Landrace | 1977 |
| BGM-0375 | 3 | North East Brazil | Ceará | Landrace | 1977 |
| BGM-0376 | 3 | North East Brazil | Ceará | Landrace | 1977 |
| BGM-0619 | 3 | North East Brazil | Ceará | Landrace | 1980 |
| BGM-0623 | 3 | North East Brazil | Ceará | Landrace | 1980 |
| BGM-0636 | 3 | North East Brazil | Ceará | Landrace | 1980 |
| BGM-0642 | 3 | North East Brazil | Ceará | Landrace | 1981 |
| BGM-0928 | 3 | Midwest Brazil | Distrito Federal | Improved variety | 1987 |
| BGM-1729 | 3 | North East Brazil | Maranhão | Landrace | 2001 |
| BGM-1738 | 3 | North East Brazil | Maranhão | Landrace | 2001 |
| BGM-1763 | 3 | North East Brazil | Maranhão | Landrace | 2001 |
| BGM-1764 | 3 | North East Brazil | Maranhão | Landrace | 2001 |
| BGM-0659 | 3 | North East Brazil | Maranhão | Landrace | 1981 |
| BGM-0501 | 3 | Southeast Brazil | Minas Gerais | Landrace | 1977 |
| BGM-0505 | 3 | Southeast Brazil | Minas Gerais | Landrace | 1977 |
| BGM-0510 | 3 | Southeast Brazil | Minas Gerais | Landrace | 1977 |
| BGM-0668 | 3 | Southeast Brazil | Minas Gerais | Landrace | 1980 |
| BGM-0890 | 3 | Venezuela | NA | Landrace | 1982 |
| BGM-1254 | 3 | Nigeria | NA | Improved variety | 1989 |
| BGM-1257 | 3 | Nigeria | NA | Improved variety | 1989 |
| BGM-0887 | 3 | Panama | NA | Improved variety | 1982 |
| BGM-1010 | 3 | Brazil-NA | NA | Landrace | 1988 |
| BGM-1180 | 3 | Brazil-NA | NA | Landrace | 1988 |
| BGM-1226 | 3 | Brazil-NA | NA | Landrace | 1989 |
| BGM-0285 | 3 | Brazil-NA | NA | Landrace | 1972 |
| BGM-0419 | 3 | Brazil-NA | NA | Landrace | 1977 |
| BGM-0443 | 3 | Brazil-NA | NA | Landrace | 1977 |
| BGM-0445 | 3 | Brazil-NA | NA | Landrace | 1977 |
| BGM-0463 | 3 | Brazil-NA | NA | Landrace | 1977 |
| BGM-0473 | 3 | Brazil-NA | NA | Landrace | 1977 |
| BGM-0631 | 3 | Brazil-NA | NA | Landrace | 1980 |
| BGM-0654 | 3 | Brazil-NA | NA | Landrace | 1981 |
| BGM-0745 | 3 | Brazil-NA | NA | Landrace | 1981 |
| BGM-0089 | 3 | Brazil-NA | NA | Landrace | 1973 |
| BGM-1196 | 3 | North Brazil | Pará | Landrace | 1989 |
| BGM-1444 | 3 | North Brazil | Pará | Landrace | 1991 |
| BGM-1668 | 3 | North Brazil | Pará | Landrace | 1999 |
| BGM-1679 | 3 | North Brazil | Pará | Landrace | 1999 |
| BGM-1332 | 3 | North East Brazil | Paraíba | Landrace | 1991 |
| BGM-1355 | 3 | North East Brazil | Paraíba | Landrace | 1991 |
| BGM-1358 | 3 | North East Brazil | Paraíba | Landrace | 1991 |
| BGM-1366 | 3 | North East Brazil | Paraíba | Landrace | 1991 |
| BGM-1662 | 3 | South Brazil | Paraná | Improved variety | 1997 |
| BGM-0930 | 3 | South Brazil | Paraná | Landrace | 1986 |
| BGM-1451 | 3 | South Brazil | Paraná | Landrace | 1991 |
| BGM-1453 | 3 | South Brazil | Paraná | Landrace | 1991 |
| BGM-1277 | 3 | North East Brazil | Pernambuco | Landrace | 1991 |
| BGM-1279 | 3 | North East Brazil | Pernambuco | Landrace | 1991 |
| BGM-1287 | 3 | North East Brazil | Pernambuco | Landrace | 1991 |
| BGM-1294 | 3 | North East Brazil | Pernambuco | Landrace | 1991 |
| BGM-1306 | 3 | North East Brazil | Pernambuco | Landrace | 1991 |
| BGM-1423 | 3 | North East Brazil | Pernambuco | Landrace | 1991 |
| BGM-1429 | 3 | North East Brazil | Pernambuco | Landrace | 1991 |
| BGM-1436 | 3 | North East Brazil | Pernambuco | Landrace | 1991 |
| BGM-1437 | 3 | North East Brazil | Pernambuco | Landrace | 1991 |
| BGM-1629 | 3 | North East Brazil | Pernambuco | Landrace | 1995 |
| BGM-1690 | 3 | North East Brazil | Pernambuco | Landrace | 2000 |
| BGM-0574 | 3 | North East Brazil | Pernambuco | Landrace | 1978 |
| BGM-0576 | 3 | North East Brazil | Pernambuco | Landrace | 1978 |
| BGM-0590 | 3 | North East Brazil | Pernambuco | Landrace | 1978 |
| BGM-1584 | 3 | North East Brazil | Piauí | Landrace | 1994 |
| BGM-1569 | 3 | North East Brazil | Piauí | Landrace | 1994 |
| BGM-1579 | 3 | North East Brazil | Piauí | Landrace | 1994 |
| BGM-1607 | 3 | North East Brazil | Piauí | Landrace | 1994 |
| BGM-1608 | 3 | North East Brazil | Piauí | Landrace | 1994 |
| BGM-1610 | 3 | North East Brazil | Piauí | Landrace | 1994 |
| BGM-0149 | 3 | Southeast Brazil | Rio de Janeiro | Landrace | 1972 |
| BGM-0436 | 3 | Southeast Brazil | Rio de Janeiro | Landrace | 1977 |
| BGM-1050 | 3 | North East Brazil | Rio Grande do Norte | Landrace | 1987 |
| BGM-1053 | 3 | North East Brazil | Rio Grande do Norte | Landrace | 1987 |
| BGM-1055 | 3 | North East Brazil | Rio Grande do Norte | Landrace | 1987 |
| BGM-1057 | 3 | North East Brazil | Rio Grande do Norte | Landrace | 1987 |
| BGM-1413 | 3 | North East Brazil | Rio Grande do Norte | Landrace | 1991 |
| BGM-2052 | 3 | South Brazil | Rio Grande do Sul | Improved variety | 2011 |
| BGM-0591 | 3 | South Brazil | Rio Grande do Sul | Improved variety | 1978 |
| BGM-1110 | 3 | South Brazil | Santa Catarina | Landrace | 1987 |
| BGM-0211 | 3 | Southeast Brazil | São Paulo | Improved variety | 1971 |
| BGM-0356 | 3 | Southeast Brazil | São Paulo | Improved variety | 1977 |
| BGM-0444 | 3 | Southeast Brazil | São Paulo | Improved variety | 1977 |
| BGM-1635 | 3 | North East Brazil | Sergipe | Landrace | 1995 |
| BGM-0260 | 3 | North East Brazil | Sergipe | Landrace | 1969 |
| BGM-0280 | 3 | North East Brazil | Sergipe | Landrace | 1972 |
| BGM-0821 | 3 | North East Brazil | Sergipe | Landrace | 1981 |
| BGM-0085 | 3 | North East Brazil | Sergipe | Landrace | 1969 |
| BGM-0856 | 3 | North East Brazil | Sergipe | Landrace | 1981 |
| BGM-0307 | 3 | Colombia | Valle | Improved Variety | 1976 |
| BGM-0870 | 3 | Colombia | Valle | Improved Variety | 1982 |
| BGM-0884 | 3 | Colombia | ValledelCauca | Improved variety | 1982 |
| BGM-0995 | 3 | Colombia | ValledelCauca | Improved variety | 1987 |
| BGM-0998 | 3 | Colombia | ValledelCauca | Improved variety | 1987 |
| BGM-0304 | 3 | Colombia | ValledelCauca | Improved variety | 1976 |
| BGM-0336 | 3 | Colombia | ValledelCauca | Improved variety | 1976 |
| BGM-0341 | 3 | Colombia | ValledelCauca | Improved variety | 1976 |
| BGM-0349 | 3 | Colombia | ValledelCauca | Improved variety | 1976 |
| BGM-0894 | 3 | Colombia | ValledelCauca | Improved variety | 1982 |
| BGM-0913 | 4 | North Brazil | Acre | Landrace | 1987 |
| BGM-0546 | 4 | North East Brazil | Alagoas | Landrace | 1978 |
| BGM-0583 | 4 | North East Brazil | Alagoas | Landrace | 1978 |
| BGM-1707 | 4 | North Brazil | Amazonas | Improved variety | 2001 |
| BGM-1715 | 4 | North Brazil | Amazonas | Improved variety | 2001 |
| BGM-0947 | 4 | North Brazil | Amazonas | Landrace | 1987 |
| BGM-1134 | 4 | North Brazil | Amazonas | Landrace | 1988 |
| BGM-1136 | 4 | North Brazil | Amazonas | Landrace | 1988 |
| BGM-1148 | 4 | North Brazil | Amazonas | Landrace | 1988 |
| BGM-1153 | 4 | North Brazil | Amazonas | Landrace | 1988 |
| BRS Gema de Ovo | 4 | North Brazil | Amazonas | Landrace | 1988 |
| 7734_7wx | 4 | North East Brazil | Bahia | Improved variety | 2010 |
| BGM-2042 | 4 | North East Brazil | Bahia | Improved Variety | 2009 |
| BRS Caipira | 4 | North East Brazil | Bahia | Improved variety | 2017 |
| BRS Tapioqueira | 4 | North East Brazil | Bahia | Improved Variety | 2009 |
| BGM-0100 | 4 | North East Brazil | Bahia | Improved variety | 1972 |
| BGM-0011 | 4 | North East Brazil | Bahia | Improved variety | 1965 |
| BGM-0160 | 4 | North East Brazil | Bahia | Improved Variety | 1972 |
| BGM-1656 | 4 | North East Brazil | Bahia | Improved variety | 1996 |
| BGM-1658 | 4 | North East Brazil | Bahia | Improved variety | 1996 |
| BGM-1723 | 4 | North East Brazil | Bahia | Improved variety | 2001 |
| BGM-1724 | 4 | North East Brazil | Bahia | Improved variety | 2001 |
| BGM-0207 | 4 | North East Brazil | Bahia | Improved variety | 1974 |
| BGM-0220 | 4 | North East Brazil | Bahia | Improved variety | 1972 |
| BGM-0232 | 4 | North East Brazil | Bahia | Improved variety | 1974 |
| BGM-0237 | 4 | North East Brazil | Bahia | Improved variety | 1974 |
| BGM-0097 | 4 | North East Brazil | Bahia | Improved variety | 1972 |
| BRS Poti Branca | 4 | North East Brazil | Bahia | Improved variety | 1987 |
| BGM-0001 | 4 | North East Brazil | Bahia | Landrace | 1962 |
| BGM-0023 | 4 | North East Brazil | Bahia | Landrace | 1969 |
| BGM-0125 | 4 | North East Brazil | Bahia | Landrace | 1972 |
| BGM-0195 | 4 | North East Brazil | Bahia | Landrace | 1972 |
| BGM-0922 | 4 | North East Brazil | Bahia | Landrace | 1983 |
| BGM-0924 | 4 | North East Brazil | Bahia | Landrace | 1983 |
| BGM-1028 | 4 | North East Brazil | Bahia | Landrace | 1987 |
| BGM-1031 | 4 | North East Brazil | Bahia | Landrace | 1987 |
| BGM-1124 | 4 | North East Brazil | Bahia | Landrace | 1987 |
| BGM-0068 | 4 | North East Brazil | Bahia | Landrace | 1972 |
| **Cigana Preta** | 4 | North East Brazil | Bahia | Landrace | 1962 |
| BGM-0010 | 4 | North East Brazil | Bahia | Landrace | 1965 |
| BGM-0103 | 4 | North East Brazil | Bahia | Landrace | 1972 |
| BGM-1085 | 4 | North East Brazil | Bahia | Landrace | 1987 |
| BGM-1087 | 4 | North East Brazil | Bahia | Landrace | 1987 |
| BGM-1123 | 4 | North East Brazil | Bahia | Landrace | 1987 |
| BGM-1126 | 4 | North East Brazil | Bahia | Landrace | 1987 |
| BGM-0115 | 4 | North East Brazil | Bahia | Landrace | 1969 |
| BGM-0116 | 4 | North East Brazil | Bahia | Landrace | 1962 |
| BGM-0120 | 4 | North East Brazil | Bahia | Landrace | 1972 |
| BGM-0127 | 4 | North East Brazil | Bahia | Landrace | 1972 |
| BGM-0130 | 4 | North East Brazil | Bahia | Landrace | 1972 |
| BGM-0136 | 4 | North East Brazil | Bahia | Landrace | 1962 |
| BGM-0138 | 4 | North East Brazil | Bahia | Landrace | 1972 |
| BGM-0146 | 4 | North East Brazil | Bahia | Landrace | 1969 |
| BGM-1481 | 4 | North East Brazil | Bahia | Landrace | 1994 |
| BGM-1487 | 4 | North East Brazil | Bahia | Landrace | 1994 |
| BGM-1489 | 4 | North East Brazil | Bahia | Landrace | 1994 |
| BGM-1495 | 4 | North East Brazil | Bahia | Landrace | 1994 |
| BGM-1498 | 4 | North East Brazil | Bahia | Landrace | 1994 |
| BGM-1499 | 4 | North East Brazil | Bahia | Landrace | 1994 |
| BGM-0150 | 4 | North East Brazil | Bahia | Landrace | 1972 |
| BGM-1503 | 4 | North East Brazil | Bahia | Landrace | 1994 |
| BGM-1513 | 4 | North East Brazil | Bahia | Landrace | 1994 |
| BGM-1515 | 4 | North East Brazil | Bahia | Landrace | 1994 |
| BGM-1518 | 4 | North East Brazil | Bahia | Landrace | 1994 |
| BGM-0152 | 4 | North East Brazil | Bahia | Landrace | 1972 |
| BGM-1520 | 4 | North East Brazil | Bahia | Landrace | 1994 |
| BGM-1535 | 4 | North East Brazil | Bahia | Landrace | 1994 |
| BGM-0155 | 4 | North East Brazil | Bahia | Landrace | 1972 |
| BGM-0156 | 4 | North East Brazil | Bahia | Landrace | 1972 |
| BGM-1560 | 4 | North East Brazil | Bahia | Landrace | 1994 |
| BGM-0162 | 4 | North East Brazil | Bahia | Landrace | 1977 |
| BGM-0164 | 4 | North East Brazil | Bahia | Landrace | 1969 |
| BGM-1643 | 4 | North East Brazil | Bahia | Landrace | 1996 |
| BGM-1649 | 4 | North East Brazil | Bahia | Landrace | 1996 |
| BGM-0166 | 4 | North East Brazil | Bahia | Landrace | 1969 |
| BGM-0167 | 4 | North East Brazil | Bahia | Landrace | 1969 |
| BGM-1681 | 4 | North East Brazil | Bahia | Landrace | 1999 |
| BGM-1684 | 4 | North East Brazil | Bahia | Landrace | 1999 |
| BGM-1688 | 4 | North East Brazil | Bahia | Landrace | 1999 |
| BGM-0169 | 4 | North East Brazil | Bahia | Landrace | 1972 |
| BGM-1691 | 4 | North East Brazil | Bahia | Landrace | 2000 |
| BGM-0178 | 4 | North East Brazil | Bahia | Landrace | 1972 |
| BGM-0181 | 4 | North East Brazil | Bahia | Landrace | 1972 |
| BGM-1814 | 4 | North East Brazil | Bahia | Landrace | 2003 |
| BGM-0182 | 4 | North East Brazil | Bahia | Landrace | 1972 |
| BGM-0185 | 4 | North East Brazil | Bahia | Landrace | 1969 |
| BGM-0208 | 4 | North East Brazil | Bahia | Landrace | 1972 |
| BGM-0021 | 4 | North East Brazil | Bahia | Landrace | 1972 |
| BGM-0217 | 4 | North East Brazil | Bahia | Landrace | 1971 |
| BGM-0242 | 4 | North East Brazil | Bahia | Landrace | 1974 |
| BGM-0025 | 4 | North East Brazil | Bahia | Landrace | 1969 |
| BGM-0261 | 4 | North East Brazil | Bahia | Landrace | 1972 |
| BGM-0266 | 4 | North East Brazil | Bahia | Landrace | 1972 |
| BGM-0271 | 4 | North East Brazil | Bahia | Landrace | 1972 |
| BGM-0273 | 4 | North East Brazil | Bahia | Landrace | 1971 |
| BGM-0028 | 4 | North East Brazil | Bahia | Landrace | 1972 |
| BGM-0281 | 4 | North East Brazil | Bahia | Landrace | 1972 |
| BGM-0282 | 4 | North East Brazil | Bahia | Landrace | 1969 |
| BGM-0033 | 4 | North East Brazil | Bahia | Landrace | 1972 |
| BGM-0359 | 4 | North East Brazil | Bahia | Landrace | 1977 |
| BGM-0047 | 4 | North East Brazil | Bahia | Landrace | 1971 |
| BGM-0048 | 4 | North East Brazil | Bahia | Landrace | 1965 |
| BGM-0556 | 4 | North East Brazil | Bahia | Landrace | 1978 |
| BGM-0569 | 4 | North East Brazil | Bahia | Landrace | 1978 |
| BGM-0069 | 4 | North East Brazil | Bahia | Landrace | 1972 |
| BGM-0767 | 4 | North East Brazil | Bahia | Landrace | 1981 |
| BGM-0778 | 4 | North East Brazil | Bahia | Landrace | 1981 |
| BGM-0785 | 4 | North East Brazil | Bahia | Landrace | 1981 |
| BGM-0788 | 4 | North East Brazil | Bahia | Landrace | 1981 |
| BGM-0791 | 4 | North East Brazil | Bahia | Landrace | 1981 |
| BGM-0799 | 4 | North East Brazil | Bahia | Landrace | 1981 |
| BGM-0083 | 4 | North East Brazil | Bahia | Landrace | 1969 |
| BGM-0868 | 4 | North East Brazil | Bahia | Landrace | 1982 |
| BGM-0088 | 4 | North East Brazil | Bahia | Landrace | 1972 |
| Corrente | 4 | North East Brazil | Bahia | Landrace | 2012 |
| BGM-0368 | 4 | North East Brazil | Ceará | Landrace | 1977 |
| BGM-0609 | 4 | North East Brazil | Ceará | Landrace | 1980 |
| BGM-1310 | 4 | North East Brazil | Ceará | Landrace | 1991 |
| BGM-0367 | 4 | North East Brazil | Ceará | Landrace | 1977 |
| BGM-0371 | 4 | North East Brazil | Ceará | Landrace | 1977 |
| BGM-0378 | 4 | North East Brazil | Ceará | Landrace | 1977 |
| BGM-0382 | 4 | North East Brazil | Ceará | Landrace | 1977 |
| BGM-0615 | 4 | North East Brazil | Ceará | Landrace | 1980 |
| BGM-0638 | 4 | North East Brazil | Ceará | Landrace | 1980 |
| BGM-0390 | 4 | Southeast Brazil | Espírito Santo | Landrace | 1977 |
| BGM-0395 | 4 | Southeast Brazil | Espírito Santo | Landrace | 1977 |
| BGM-0399 | 4 | Southeast Brazil | Espírito Santo | Landrace | 1977 |
| BGM-0405 | 4 | Southeast Brazil | Espírito Santo | Landrace | 1971 |
| BGM-0455 | 4 | Southeast Brazil | Espírito Santo | Landrace | 1977 |
| BGM-1737 | 4 | North East Brazil | Maranhão | Landrace | 2001 |
| BGM-1750 | 4 | North East Brazil | Maranhão | Landrace | 2001 |
| BGM-0671 | 4 | North East Brazil | Maranhão | Landrace | 1981 |
| BGM-1100 | 4 | Midwest Brazil | Mato Grosso | Landrace | 1987 |
| BGM-0657 | 4 | Midwest Brazil | Mato Grosso | Landrace | 1981 |
| BGM-0128 | 4 | Southeast Brazil | Minas Gerais | Landrace | 1972 |
| BGM-0503 | 4 | Southeast Brazil | Minas Gerais | Landrace | 1977 |
| BGM-0523 | 4 | Southeast Brazil | Minas Gerais | Landrace | 1977 |
| BGM-1259 | 4 | Nigeria | NA | Improved variety | 1989 |
| BGM-0343 | 4 | Venezuela | NA | Improved variety | 1976 |
| BGM-0460 | 4 | Brazil-NA | NA | Improved Variety | 1977 |
| BGM-0374 | 4 | Brazil-NA | NA | Landrace | 1977 |
| BGM-0433 | 4 | Brazil-NA | NA | Landrace | 1977 |
| BGM-1253 | 4 | Nigeria | NA | Landrace | 1989 |
| BGM-1103 | 4 | Brazil-NA | NA | Landrace | 1987 |
| BGM-1156 | 4 | Brazil-NA | NA | Landrace | 1988 |
| BGM-1163 | 4 | Brazil-NA | NA | Landrace | 1988 |
| BGM-1167 | 4 | Brazil-NA | NA | Landrace | 1988 |
| BGM-0124 | 4 | Brazil-NA | NA | Landrace | 1972 |
| BGM-0143 | 4 | Brazil-NA | NA | Landrace | 1972 |
| BGM-0144 | 4 | Brazil-NA | NA | Landrace | 1972 |
| BGM-0148 | 4 | Brazil-NA | NA | Landrace | 1972 |
| BGM-0157 | 4 | Brazil-NA | NA | Landrace | 1972 |
| BGM-0165 | 4 | Brazil-NA | NA | Landrace | 1972 |
| BGM-0171 | 4 | Brazil-NA | NA | Landrace | 1972 |
| BGM-0174 | 4 | Brazil-NA | NA | Landrace | 1973 |
| BGM-0183 | 4 | Brazil-NA | NA | Landrace | 1973 |
| BGM-0238 | 4 | Brazil-NA | NA | Landrace | 1974 |
| BGM-0283 | 4 | Brazil-NA | NA | Landrace | 1972 |
| BGM-0287 | 4 | Brazil-NA | NA | Landrace | 1972 |
| BGM-0031 | 4 | Brazil-NA | NA | Landrace | 1973 |
| BGM-0361 | 4 | Brazil-NA | NA | Landrace | 1977 |
| BGM-0037 | 4 | Brazil-NA | NA | Landrace | 1972 |
| BGM-0041 | 4 | Brazil-NA | NA | Landrace | 1971 |
| BGM-0432 | 4 | Brazil-NA | NA | Landrace | 1977 |
| BGM-0434 | 4 | Brazil-NA | NA | Landrace | 1977 |
| BGM-0438 | 4 | Brazil-NA | NA | Landrace | 1977 |
| BGM-0447 | 4 | Brazil-NA | NA | Landrace | 1977 |
| BGM-0045 | 4 | Brazil-NA | NA | Landrace | 1971 |
| BGM-0452 | 4 | Brazil-NA | NA | Landrace | 1977 |
| BGM-0456 | 4 | Brazil-NA | NA | Landrace | 1977 |
| BGM-0462 | 4 | Brazil-NA | NA | Landrace | 1977 |
| BGM-0469 | 4 | Brazil-NA | NA | Landrace | 1977 |
| BGM-0478 | 4 | Brazil-NA | NA | Landrace | 1977 |
| BGM-0484 | 4 | Brazil-NA | NA | Landrace | 1977 |
| BGM-0489 | 4 | Brazil-NA | NA | Landrace | 1977 |
| BGM-0498 | 4 | Brazil-NA | NA | Landrace | 1977 |
| BGM-0499 | 4 | Brazil-NA | NA | Landrace | 1977 |
| BGM-0575 | 4 | Brazil-NA | NA | Landrace | 1978 |
| BGM-0075 | 4 | Brazil-NA | NA | Landrace | 1973 |
| BGM-0096 | 4 | Brazil-NA | NA | Landrace | 1972 |
| BGM-0098 | 4 | Brazil-NA | NA | Landrace | 1973 |
| BGM-1835 | 4 | North Brazil | Pará | Improved variety | 2005 |
| BGM-0551 | 4 | North Brazil | Pará | Improved variety | 1978 |
| BGM-1670 | 4 | North Brazil | Pará | Landrace | 1999 |
| BGM-1671 | 4 | North Brazil | Pará | Landrace | 1999 |
| BGM-1667 | 4 | North Brazil | Pará | Landrace | 1999 |
| BGM-1669 | 4 | North Brazil | Pará | Landrace | 1999 |
| BGM-1672 | 4 | North Brazil | Pará | Landrace | 1999 |
| BGM-1680 | 4 | North Brazil | Pará | Landrace | 1999 |
| BGM-0567 | 4 | North Brazil | Pará | Landrace | 1978 |
| BGM-1361 | 4 | North East Brazil | Paraíba | Landrace | 1991 |
| BGM-1370 | 4 | North East Brazil | Paraíba | Landrace | 1991 |
| BGM-1079 | 4 | North East Brazil | Paraíba | Landrace | 1987 |
| BGM-1327 | 4 | North East Brazil | Paraíba | Landrace | 1991 |
| BGM-1333 | 4 | North East Brazil | Paraíba | Landrace | 1991 |
| BGM-1359 | 4 | North East Brazil | Paraíba | Landrace | 1991 |
| BGM-1362 | 4 | North East Brazil | Paraíba | Landrace | 1991 |
| BGM-1368 | 4 | North East Brazil | Paraíba | Landrace | 1991 |
| BGM-1371 | 4 | North East Brazil | Paraíba | Landrace | 1991 |
| BGM-1621 | 4 | North East Brazil | Paraíba | Landrace | 1995 |
| BGM-1454 | 4 | South Brazil | Paraná | Landrace | 1991 |
| BGM-1455 | 4 | South Brazil | Paraná | Landrace | 1991 |
| BGM-1081 | 4 | North East Brazil | Pernambuco | Landrace | 1987 |
| BGM-1281 | 4 | North East Brazil | Pernambuco | Landrace | 1991 |
| BGM-1291 | 4 | North East Brazil | Pernambuco | Landrace | 1991 |
| BGM-1296 | 4 | North East Brazil | Pernambuco | Landrace | 1991 |
| BGM-1313 | 4 | North East Brazil | Pernambuco | Landrace | 1991 |
| BGM-1315 | 4 | North East Brazil | Pernambuco | Landrace | 1991 |
| BGM-1321 | 4 | North East Brazil | Pernambuco | Landrace | 1991 |
| BGM-0539 | 4 | North East Brazil | Pernambuco | Landrace | 1977 |
| BGM-0549 | 4 | North East Brazil | Pernambuco | Landrace | 1978 |
| BGM-0579 | 4 | North East Brazil | Pernambuco | Landrace | 1978 |
| BGM-0080 | 4 | North East Brazil | Pernambuco | Landrace | 1969 |
| BGM-1586 | 4 | North East Brazil | Piauí | Landrace | 1994 |
| BGM-0216 | 4 | Southeast Brazil | Rio de Janeiro | Improved variety | 1973 |
| BGM-0472 | 4 | Southeast Brazil | Rio de Janeiro | Improved variety | 1977 |
| BGM-0215 | 4 | Southeast Brazil | Rio de Janeiro | Landrace | 1972 |
| BGM-0389 | 4 | Southeast Brazil | Rio de Janeiro | Landrace | 1977 |
| BGM-0394 | 4 | Southeast Brazil | Rio de Janeiro | Landrace | 1977 |
| BGM-0398 | 4 | Southeast Brazil | Rio de Janeiro | Landrace | 1977 |
| BGM-1073 | 4 | North East Brazil | Rio Grande do Norte | Landrace | 1987 |
| BGM-1407 | 4 | North East Brazil | Rio Grande do Norte | Landrace | 1991 |
| BGM-1416 | 4 | North East Brazil | Rio Grande do Norte | Landrace | 1991 |
| BGM-1403 | 4 | North East Brazil | Rio Grande do Norte | Landrace | 1991 |
| BGM-1406 | 4 | North East Brazil | Rio Grande do Norte | Landrace | 1991 |
| BGM-1410 | 4 | North East Brazil | Rio Grande do Norte | Landrace | 1991 |
| BGM-1412 | 4 | North East Brazil | Rio Grande do Norte | Landrace | 1991 |
| BGM-1417 | 4 | North East Brazil | Rio Grande do Norte | Landrace | 1991 |
| BGM-0189 | 4 | North East Brazil | Rio Grande do Norte | Landrace | 1971 |
| BGM-0091 | 4 | North East Brazil | Rio Grande do Norte | Landrace | 1971 |
| BGM-0441 | 4 | South Brazil | Rio Grande do Sul | Landrace | 1977 |
| BGM-0592 | 4 | South Brazil | Rio Grande do Sul | Landrace | 1978 |
| BGM-1125 | 4 | North Brazil | Rondônia | Landrace | 1987 |
| BGM-1816 | 4 | North Brazil | Roraima | Landrace | 2003 |
| BGM-1111 | 4 | South Brazil | Santa Catarina | Landrace | 1988 |
| BGM-0082 | 4 | South Brazil | Santa Catarina | Landrace | 1971 |
| BGM-0177 | 4 | Southeast Brazil | São Paulo | Improved variety | 1972 |
| BGM-0214 | 4 | Southeast Brazil | São Paulo | Improved variety | 1977 |
| BGM-0276 | 4 | Southeast Brazil | São Paulo | Improved variety | 1972 |
| BGM-0308 | 4 | Southeast Brazil | São Paulo | Improved Variety | 1976 |
| BGM-0414 | 4 | Southeast Brazil | São Paulo | Improved variety | 1977 |
| BGM-0442 | 4 | Southeast Brazil | São Paulo | Improved variety | 1977 |
| BGM-0477 | 4 | Southeast Brazil | São Paulo | Improved variety | 1977 |
| BGM-0557 | 4 | Southeast Brazil | São Paulo | Improved Variety | 1978 |
| BGM-0024 | 4 | North East Brazil | Sergipe | Landrace | 1969 |
| BGM-0834 | 4 | North East Brazil | Sergipe | Landrace | 1981 |
| BGM-1646 | 4 | North East Brazil | Sergipe | Landrace | 1996 |
| BGM-0277 | 4 | North East Brazil | Sergipe | Landrace | 1972 |
| BGM-0822 | 4 | North East Brazil | Sergipe | Landrace | 1972 |
| BGM-0824 | 4 | North East Brazil | Sergipe | Landrace | 1981 |
| BGM-0826 | 4 | North East Brazil | Sergipe | Landrace | 1981 |
| BGM-0837 | 4 | North East Brazil | Sergipe | Landrace | 1981 |
| BGM-0846 | 4 | North East Brazil | Sergipe | Landrace | 1981 |
| BGM-0847 | 4 | North East Brazil | Sergipe | Landrace | 1981 |
| Valência | 4 | North East Brazil | Sergipe | Landrace | 1996 |
| BGM-1037 | 4 | North Brazil | Tocantins | Landrace | 1987 |
| BGM-0303 | 4 | Colombia | Valle | Improved Variety | 1976 |
| BGM-0330 | 4 | Colombia | Valle | Improved Variety | 1976 |
| BGM-0603 | 4 | Colombia | Valle | Improved Variety | 1978 |
| BGM-0891 | 4 | Colombia | ValledelCauca | Improved variety | 1982 |
| BGM-0289 | 4 | Colombia | ValledelCauca | Improved variety | 1976 |
| BGM-0295 | 4 | Colombia | ValledelCauca | Improved variety | 1976 |
| BGM-0297 | 4 | Colombia | ValledelCauca | Improved variety | 1976 |
| BGM-0298 | 4 | Colombia | ValledelCauca | Improved variety | 1976 |
| BGM-0316 | 4 | Colombia | ValledelCauca | Improved variety | 1976 |
| BGM-0318 | 4 | Colombia | ValledelCauca | Improved variety | 1976 |
| BGM-0319 | 4 | Colombia | ValledelCauca | Improved variety | 1976 |
| BGM-0324 | 4 | Colombia | ValledelCauca | Improved variety | 1976 |
| BGM-0332 | 4 | Colombia | ValledelCauca | Improved variety | 1976 |
| BGM-0338 | 4 | Colombia | ValledelCauca | Improved variety | 1976 |
| BGM-0885 | 4 | Colombia | ValledelCauca | Improved variety | 1982 |
| BGM-0702 | 5 | North Brazil | Acre | Landrace | 1981 |
| BGM-0932 | 5 | North East Brazil | Alagoas | Landrace | 1987 |
| BGM-0552 | 5 | North East Brazil | Alagoas | Landrace | 1972 |
| BGM-0807 | 5 | North East Brazil | Alagoas | Landrace | 1981 |
| BGM-0810 | 5 | North East Brazil | Alagoas | Landrace | 1981 |
| BGM-0815 | 5 | North East Brazil | Alagoas | Landrace | 1981 |
| BGM-1458 | 5 | North Brazil | Amapá | Landrace | 1992 |
| BGM-0951 | 5 | North Brazil | Amazonas | Landrace | 1987 |
| BGM-0955 | 5 | North Brazil | Amazonas | Landrace | 1987 |
| BGM-1211 | 5 | North Brazil | Amazonas | Landrace | 1989 |
| 9624-09 | 5 | North East Brazil | Bahia | Improved variety | 2010 |
| BGM-0016 | 5 | North East Brazil | Bahia | Improved variety | 1965 |
| BGM-2017 | 5 | North East Brazil | Bahia | Improved variety | 2007 |
| BGM-2043 | 5 | North East Brazil | Bahia | Improved variety | 2009 |
| BRS Verdinha | 5 | North East Brazil | Bahia | Improved Variety | 2009 |
| BGM-0072 | 5 | North East Brazil | Bahia | Landrace | 1962 |
| BGM-0205 | 5 | North East Brazil | Bahia | Landrace | 1969 |
| BGM-0934 | 5 | North East Brazil | Bahia | Landrace | 1987 |
| BGM-0937 | 5 | North East Brazil | Bahia | Landrace | 1987 |
| BGM-1026 | 5 | North East Brazil | Bahia | Landrace | 1987 |
| BGM-1027 | 5 | North East Brazil | Bahia | Landrace | 1987 |
| BGM-1029 | 5 | North East Brazil | Bahia | Landrace | 1987 |
| BGM-1512 | 5 | North East Brazil | Bahia | Landrace | 1994 |
| BGM-1116 | 5 | North East Brazil | Bahia | Landrace | 1988 |
| BGM-1200 | 5 | North East Brazil | Bahia | Landrace | 1989 |
| BGM-1202 | 5 | North East Brazil | Bahia | Landrace | 1989 |
| BGM-0121 | 5 | North East Brazil | Bahia | Landrace | 1972 |
| BGM-0126 | 5 | North East Brazil | Bahia | Landrace | 1972 |
| BGM-0135 | 5 | North East Brazil | Bahia | Landrace | 1962 |
| BGM-0137 | 5 | North East Brazil | Bahia | Landrace | 1972 |
| BGM-1440 | 5 | North East Brazil | Bahia | Landrace | 1991 |
| BGM-1476 | 5 | North East Brazil | Bahia | Landrace | 1994 |
| BGM-1482 | 5 | North East Brazil | Bahia | Landrace | 1994 |
| BGM-1484 | 5 | North East Brazil | Bahia | Landrace | 1994 |
| BGM-0151 | 5 | North East Brazil | Bahia | Landrace | 1972 |
| BGM-1516 | 5 | North East Brazil | Bahia | Landrace | 1994 |
| BGM-1521 | 5 | North East Brazil | Bahia | Landrace | 1994 |
| BGM-1545 | 5 | North East Brazil | Bahia | Landrace | 1994 |
| BGM-0163 | 5 | North East Brazil | Bahia | Landrace | 1972 |
| BGM-1640 | 5 | North East Brazil | Bahia | Landrace | 1995 |
| BGM-1645 | 5 | North East Brazil | Bahia | Landrace | 1996 |
| BGM-0179 | 5 | North East Brazil | Bahia | Landrace | 1972 |
| BGM-0184 | 5 | North East Brazil | Bahia | Landrace | 1969 |
| BGM-0186 | 5 | North East Brazil | Bahia | Landrace | 1972 |
| BGM-0249 | 5 | North East Brazil | Bahia | Landrace | 1962 |
| BGM-0263 | 5 | North East Brazil | Bahia | Landrace | 1972 |
| BGM-0029 | 5 | North East Brazil | Bahia | Landrace | 1972 |
| BGM-0036 | 5 | North East Brazil | Bahia | Landrace | 1971 |
| BGM-0039 | 5 | North East Brazil | Bahia | Landrace | 1969 |
| BGM-0040 | 5 | North East Brazil | Bahia | Landrace | 1972 |
| BGM-0042 | 5 | North East Brazil | Bahia | Landrace | 1971 |
| BGM-0051 | 5 | North East Brazil | Bahia | Landrace | 1966 |
| BGM-0053 | 5 | North East Brazil | Bahia | Landrace | 1987 |
| BGM-0541 | 5 | North East Brazil | Bahia | Landrace | 1978 |
| BGM-0563 | 5 | North East Brazil | Bahia | Landrace | 1978 |
| BGM-0076 | 5 | North East Brazil | Bahia | Landrace | 1965 |
| BGM-0781 | 5 | North East Brazil | Bahia | Landrace | 1981 |
| BGM-0783 | 5 | North East Brazil | Bahia | Landrace | 1981 |
| BGM-0867 | 5 | North East Brazil | Bahia | Landrace | 1982 |
| BGM-0090 | 5 | North East Brazil | Bahia | Landrace | 1972 |
| BGM-0604 | 5 | North East Brazil | Ceará | Landrace | 1980 |
| BGM-0611 | 5 | North East Brazil | Ceará | Landrace | 1980 |
| BGM-0649 | 5 | North East Brazil | Ceará | Landrace | 1981 |
| BGM-1044 | 5 | North East Brazil | Ceará | Landrace | 1987 |
| BGM-0650 | 5 | North East Brazil | Ceará | Landrace | 1981 |
| BGM-0383 | 5 | North East Brazil | Ceará | Landrace | 1977 |
| BGM-0384 | 5 | North East Brazil | Ceará | Landrace | 1977 |
| BGM-0618 | 5 | North East Brazil | Ceará | Landrace | 1980 |
| BGM-0620 | 5 | North East Brazil | Ceará | Landrace | 1980 |
| BGM-0630 | 5 | North East Brazil | Ceará | Landrace | 1980 |
| BGM-0635 | 5 | North East Brazil | Ceará | Landrace | 1980 |
| BGM-0640 | 5 | North East Brazil | Ceará | Landrace | 1980 |
| BGM-0645 | 5 | North East Brazil | Ceará | Landrace | 1981 |
| BGM-0655 | 5 | North East Brazil | Ceará | Landrace | 1981 |
| BGM-0901 | 5 | North East Brazil | Ceará | Landrace | 1982 |
| BGM-0388 | 5 | Southeast Brazil | Espírito Santo | Landrace | 1977 |
| BGM-0400 | 5 | Southeast Brazil | Espírito Santo | Landrace | 1981 |
| BGM-0406 | 5 | Southeast Brazil | Espírito Santo | Landrace | 1977 |
| BGM-1755 | 5 | North East Brazil | Maranhão | Landrace | 2001 |
| BGM-0665 | 5 | North East Brazil | Maranhão | Landrace | 1981 |
| BGM-0694 | 5 | North East Brazil | Maranhão | Landrace | 1981 |
| BGM-0697 | 5 | North East Brazil | Maranhão | Landrace | 1981 |
| BGM-0512 | 5 | Southeast Brazil | Minas Gerais | Landrace | 1977 |
| BGM-0520 | 5 | Southeast Brazil | Minas Gerais | Landrace | 1977 |
| BGM-0892 | 5 | Southeast Brazil | Minas Gerais | Landrace | 1982 |
| BGM-0320 | 5 | Mexico | NA | Improved Variety | 1976 |
| BGM-1245 | 5 | Nigeria | NA | Improved variety | 1989 |
| BGM-1619 | 5 | Brazil-NA | NA | Improved variety | 1995 |
| BGM-0426 | 5 | Brazil-NA | NA | Improved variety | 1977 |
| BGM-0532 | 5 | Venezuela | NA | Improved Variety | 1976 |
| BGM-0652 | 5 | Brazil-NA | NA | Landrace | 1981 |
| BGM-1155 | 5 | Brazil-NA | NA | Landrace | 1988 |
| BGM-1174 | 5 | Brazil-NA | NA | Landrace | 1988 |
| BGM-1175 | 5 | Brazil-NA | NA | Landrace | 1988 |
| BGM-1194 | 5 | Brazil-NA | NA | Landrace | 1989 |
| BGM-1195 | 5 | Brazil-NA | NA | Landrace | 1989 |
| BGM-1217 | 5 | Brazil-NA | NA | Landrace | 1989 |
| BGM-1219 | 5 | Brazil-NA | NA | Landrace | 1987 |
| BGM-0022 | 5 | Brazil-NA | NA | Landrace | 1972 |
| BGM-0247 | 5 | Brazil-NA | NA | Landrace | 1974 |
| BGM-0027 | 5 | Brazil-NA | NA | Landrace | 1969 |
| BGM-0366 | 5 | Brazil-NA | NA | Landrace | 1977 |
| BGM-0408 | 5 | Brazil-NA | NA | Landrace | 1977 |
| BGM-0411 | 5 | Brazil-NA | NA | Landrace | 1977 |
| BGM-0420 | 5 | Brazil-NA | NA | Landrace | 1977 |
| BGM-0427 | 5 | Brazil-NA | NA | Landrace | 1977 |
| BGM-0435 | 5 | Brazil-NA | NA | Landrace | 1977 |
| BGM-0439 | 5 | Brazil-NA | NA | Landrace | 1977 |
| BGM-0044 | 5 | Brazil-NA | NA | Landrace | 1971 |
| BGM-0465 | 5 | Brazil-NA | NA | Landrace | 1977 |
| BGM-0471 | 5 | Brazil-NA | NA | Landrace | 1977 |
| BGM-0474 | 5 | Brazil-NA | NA | Landrace | 1977 |
| BGM-0049 | 5 | Brazil-NA | NA | Landrace | 1973 |
| BGM-0492 | 5 | Brazil-NA | NA | Landrace | 1977 |
| BGM-0504 | 5 | Brazil-NA | NA | Landrace | 1977 |
| BGM-0052 | 5 | Brazil-NA | NA | Landrace | 1972 |
| BGM-0589 | 5 | Brazil-NA | NA | Landrace | 1978 |
| BGM-0060 | 5 | Brazil-NA | NA | Landrace | 1972 |
| BGM-0608 | 5 | Brazil-NA | NA | Landrace | 1980 |
| BGM-0093 | 5 | Brazil-NA | NA | Landrace | 1973 |
| BGM-1850 | 5 | North Brazil | Pará | Improved variety | 2005 |
| BGM-0957 | 5 | North Brazil | Pará | Landrace | 1987 |
| BGM-1165 | 5 | North Brazil | Pará | Landrace | 1988 |
| BGM-1186 | 5 | North Brazil | Pará | Landrace | 1989 |
| BGM-1638 | 5 | North Brazil | Pará | Landrace | 1995 |
| BGM-0876 | 5 | North Brazil | Pará | Landrace | 1982 |
| BGM-1077 | 5 | North East Brazil | Paraíba | Landrace | 1987 |
| BGM-1078 | 5 | North East Brazil | Paraíba | Landrace | 1987 |
| BGM-1122 | 5 | North East Brazil | Paraíba | Landrace | 1987 |
| BGM-1348 | 5 | North East Brazil | Paraíba | Landrace | 1991 |
| BGM-1345 | 5 | North East Brazil | Paraíba | Landrace | 1991 |
| BGM-1347 | 5 | North East Brazil | Paraíba | Landrace | 1991 |
| BGM-1363 | 5 | North East Brazil | Paraíba | Landrace | 1991 |
| BGM-1369 | 5 | North East Brazil | Paraíba | Landrace | 1991 |
| BGM-1376 | 5 | North East Brazil | Paraíba | Landrace | 1991 |
| BGM-1382 | 5 | North East Brazil | Paraíba | Landrace | 1991 |
| BGM-1383 | 5 | North East Brazil | Paraíba | Landrace | 1991 |
| BGM-1626 | 5 | North East Brazil | Paraíba | Landrace | 1995 |
| BGM-1452 | 5 | South Brazil | Paraná | Landrace | 1991 |
| BGM-0573 | 5 | South Brazil | Paraná | Landrace | 1978 |
| BGM-1298 | 5 | North East Brazil | Pernambuco | Landrace | 1991 |
| BGM-1262 | 5 | North East Brazil | Pernambuco | Landrace | 1991 |
| BGM-1265 | 5 | North East Brazil | Pernambuco | Landrace | 1991 |
| BGM-1267 | 5 | North East Brazil | Pernambuco | Landrace | 1991 |
| BGM-1273 | 5 | North East Brazil | Pernambuco | Landrace | 1991 |
| BGM-1282 | 5 | North East Brazil | Pernambuco | Landrace | 1991 |
| BGM-1283 | 5 | North East Brazil | Pernambuco | Landrace | 1991 |
| BGM-1293 | 5 | North East Brazil | Pernambuco | Landrace | 1991 |
| BGM-1303 | 5 | North East Brazil | Pernambuco | Landrace | 1991 |
| BGM-1305 | 5 | North East Brazil | Pernambuco | Landrace | 1991 |
| BGM-1314 | 5 | North East Brazil | Pernambuco | Landrace | 1991 |
| BGM-1339 | 5 | North East Brazil | Pernambuco | Landrace | 1991 |
| BGM-1342 | 5 | North East Brazil | Pernambuco | Landrace | 1991 |
| BGM-1432 | 5 | North East Brazil | Pernambuco | Landrace | 1991 |
| BGM-1696 | 5 | North East Brazil | Pernambuco | Landrace | 2001 |
| BGM-0561 | 5 | North East Brazil | Pernambuco | Landrace | 1978 |
| BGM-0905 | 5 | North East Brazil | Pernambuco | Landrace | 1987 |
| BGM-0230 | 5 | North East Brazil | Pernambuco | Landrace | 1972 |
| BGM-1576 | 5 | North East Brazil | Piauí | Landrace | 1994 |
| BGM-1590 | 5 | North East Brazil | Piauí | Landrace | 1994 |
| BGM-1596 | 5 | North East Brazil | Piauí | Landrace | 1994 |
| BGM-1599 | 5 | North East Brazil | Piauí | Landrace | 1994 |
| BGM-1601 | 5 | North East Brazil | Piauí | Landrace | 1994 |
| BGM-1604 | 5 | North East Brazil | Piauí | Landrace | 1994 |
| BGM-1609 | 5 | North East Brazil | Piauí | Landrace | 1994 |
| BGM-1611 | 5 | North East Brazil | Piauí | Landrace | 1994 |
| BGM-1615 | 5 | North East Brazil | Piauí | Landrace | 1994 |
| BGM-0486 | 5 | Southeast Brazil | Rio de Janeiro | Improved Variety | 1977 |
| BGM-0454 | 5 | Southeast Brazil | Rio de Janeiro | Improved variety | 1977 |
| BGM-0521 | 5 | Southeast Brazil | Rio de Janeiro | Improved variety | 1977 |
| BGM-0670 | 5 | Southeast Brazil | Rio de Janeiro | Landrace | 1981 |
| BGM-1068 | 5 | North East Brazil | Rio Grande do Norte | Landrace | 1987 |
| BGM-1409 | 5 | North East Brazil | Rio Grande do Norte | Landrace | 1991 |
| BGM-1120 | 5 | North East Brazil | Rio Grande do Norte | Landrace | 1987 |
| BGM-1401 | 5 | North East Brazil | Rio Grande do Norte | Landrace | 1991 |
| BGM-1402 | 5 | North East Brazil | Rio Grande do Norte | Landrace | 1991 |
| BGM-1408 | 5 | North East Brazil | Rio Grande do Norte | Landrace | 1991 |
| BGM-1420 | 5 | North East Brazil | Rio Grande do Norte | Landrace | 1991 |
| BGM-0600 | 5 | South Brazil | Rio Grande do Sul | Landrace | 1978 |
| BGM-0667 | 5 | South Brazil | Rio Grande do Sul | Landrace | 1981 |
| BGM-0030 | 5 | South Brazil | Santa Catarina | Landrace | 1972 |
| BGM-0236 | 5 | Southeast Brazil | São Paulo | Improved variety | 1974 |
| BGM-0422 | 5 | Southeast Brazil | São Paulo | Improved variety | 1977 |
| BGM-0626 | 5 | Southeast Brazil | São Paulo | Landrace | 1980 |
| BGM-0639 | 5 | Southeast Brazil | São Paulo | Landrace | 1980 |
| BGM-1634 | 5 | North East Brazil | Sergipe | Landrace | 1995 |
| BGM-1655 | 5 | North East Brazil | Sergipe | Landrace | 1996 |
| BGM-0065 | 5 | North East Brazil | Sergipe | Landrace | 1972 |
| BGM-0818 | 5 | North East Brazil | Sergipe | Landrace | 1981 |
| BGM-0820 | 5 | North East Brazil | Sergipe | Landrace | 1981 |
| BGM-0823 | 5 | North East Brazil | Sergipe | Landrace | 1981 |
| BGM-0845 | 5 | North East Brazil | Sergipe | Landrace | 1981 |
| BGM-0087 | 5 | North East Brazil | Sergipe | Landrace | 1969 |
| BGM-1876 | 5 | North Brazil | Tocantins | Landrace | 2005 |
| BGM-0310 | 5 | Colombia | Valle | Improved Variety | 1976 |
| BGM-0360 | 5 | Colombia | Valle | Improved Variety | 1977 |
| BGM-0601 | 5 | Colombia | Valle | Improved Variety | 1978 |
| BGM-0906 | 5 | Colombia | Valle | Improved Variety | 1987 |
| BGM-0312 | 5 | Colombia | ValledelCauca | Improved variety | 1976 |
| BGM-0331 | 5 | Colombia | ValledelCauca | Improved variety | 1976 |
| BGM-0337 | 5 | Colombia | ValledelCauca | Improved variety | 1976 |
| BGM-0658 | 5 | Colombia | ValledelCauca | Improved variety | 1981 |
| BGM-0666 | 5 | Colombia | ValledelCauca | Improved variety | 1981 |
| BGM-0873 | 5 | Colombia | ValledelCauca | Improved variety | 1982 |
| BGM-0877 | 5 | Colombia | ValledelCauca | Improved variety | 1982 |
