# Supplementary material for: Genome-wide association study of cassava starch paste properties
Source: PLoS One. 2022 Jan 21;17(1):e0262888. doi: 10.1371/journal.pone.0262888 (PMC8782291; doi:10.1371/journal.pone.0262888)
Supplement: S2 Table — (DOCX) [file pone.0262888.s008.docx]

**Genome-wide association study of cassava starch paste properties**

**S2 Table**. Average, maximum and minimum temperature and average rainfall by month during the crop season (2015/2016 and 2016/2017).

| **Year** | **Month** | **Rainfall** | **Temperature** | | | **Relative humidity** |
| --- | --- | --- | --- | --- | --- | --- |
|  |  |  | **T. max** | **t. min** | **Tmed** |  |
| 2015 | May | 266.50 | 27.28 | 21.19 | 23.54 | 91.30 |
| 2015 | June | 222.20 | 26.36 | 20.36 | 22.66 | 92.04 |
| 2015 | July | 143.20 | 26.20 | 19.60 | 22.21 | 90.20 |
| 2015 | August | 108.10 | 26.31 | 18.83 | 21.80 | 86.90 |
| 2015 | September | 27.30 | 28.89 | 19.67 | 23.14 | 82.16 |
| 2015 | October | 14.80 | 29.94 | 20.56 | 23.76 | 79.54 |
| 2015 | November | 3.00 | 32.91 | 22.50 | 25.99 | 73.06 |
| 2015 | December | 9.60 | 33.12 | 22.56 | 26.50 | 72.20 |
| 2016 | January | 179.70 | 31.18 | 23.48 | 26.08 | 82.93 |
| 2016 | February | 51.00 | 31.55 | 22.63 | 25.74 | 79.37 |
| 2016 | March | 45.40 | 32.30 | 22.79 | 26.22 | 80.53 |
| 2016 | April | 67.90 | 30.75 | 22.14 | 25.35 | 80.62 |
| 2016 | May | 158.60 | 28.32 | 21.06 | 23.85 | 85.60 |
| 2016 | June | 83.40 | 27.31 | 20.17 | 23.04 | 82.63 |
| 2016 | July | 79.60 | 26.90 | 18.90 | 22.11 | 83.47 |
| 2016 | August | 148.90 | 27.26 | 18.92 | 22.20 | 81.47 |
| 2016 | September | 70.50 | 27.63 | 19.69 | 22.87 | 81.35 |
| 2016 | October | 60.00 | 29.66 | 21.29 | 24.46 | 82.56 |
| 2016 | November | 34.60 | 30.79 | 21.57 | 25.02 | 79.07 |
| 2016 | December | 60.00 | 31.66 | 22.27 | 25.65 | 78.45 |
| 2017 | January | 14.50 | 31.99 | 21.73 | 25.65 | 75.77 |
| 2017 | February | 40.10 | 31.66 | 22.16 | 25.86 | 78.65 |
| 2017 | March | 43.90 | 31.54 | 22.38 | 25.93 | 77.43 |
| 2017 | April | 152.60 | 29.54 | 22.17 | 24.86 | 83.93 |
| 2017 | May | 171.20 | 27.42 | 20.94 | 23.38 | 89.28 |
| 2017 | June | 135.90 | 26.79 | 20.03 | 22.66 | 85.73 |
| 2017 | July | 160.60 | 24.60 | 18.01 | 20.80 | 87.68 |
