# Supplementary material for: Genome-wide association study of cassava starch paste properties
Source: PLoS One. 2022 Jan 21;17(1):e0262888. doi: 10.1371/journal.pone.0262888 (PMC8782291; doi:10.1371/journal.pone.0262888)
Supplement: S3 Table — (DOCX) [file pone.0262888.s009.docx]

**Genome-wide association study of cassava starch paste properties**

**S3 Table**. Genomic coverage of the single nucleotide polymorphism (SNP) markers on the 18 cassava chromosomes.

| Chromosome | Chromosome size (Mb) | Number of SNPs | Mean density (kb) |
| --- | --- | --- | --- |
| 1 | 34.95 | 2133 | 16.39 |
| 2 | 32.37 | 1492 | 21.7 |
| 3 | 29.31 | 1607 | 18.24 |
| 4 | 28.73 | 1187 | 24.2 |
| 5 | 28.33 | 1436 | 19.73 |
| 6 | 27.89 | 1302 | 21.42 |
| 7 | 26.78 | 882 | 30.37 |
| 8 | 33.85 | 1107 | 30.57 |
| 9 | 29.4 | 1230 | 23.91 |
| 10 | 26.32 | 1352 | 19.46 |
| 11 | 27.27 | 1334 | 20.44 |
| 12 | 31.56 | 917 | 34.41 |
| 13 | 28.1 | 1026 | 27.39 |
| 14 | 24.5 | 1365 | 17.95 |
| 15 | 26.01 | 1298 | 20.04 |
| 16 | 28.96 | 965 | 30.01 |
| 17 | 27.36 | 1382 | 19.8 |
| 18 | 25.1 | 1063 | 23.61 |
| **Mean** | 28.71 | 1282 | 23.31 |
| **Total** | 516.79 | 23078 | - |
