# Supplementary material for: Genome-wide association study of cassava starch paste properties
Source: PLoS One. 2022 Jan 21;17(1):e0262888. doi: 10.1371/journal.pone.0262888 (PMC8782291; doi:10.1371/journal.pone.0262888)
Supplement: S4 Table — For the SNPs, the list of annotated genes and transcripts close to each window explained most of the genetic variance for cassava starch pasting properties. (DOCX) [file pone.0262888.s010.docx]

**Genome-wide association study of cassava starch paste properties**

**S4 Table**. Analysis summary of the PANTHER (PTH) and Pfam (PF) database, as well as EuKaryotic Orthologous Groups (KOG) and Gene Antology annotation. For the SNPs, the list of annotated genes and transcripts close to each window explained most of the genetic variance for cassava starch pasting properties.

| **SNP** | **pos1** | **pos2** | **Locus Name** | **Definition from PF and PTH** | **PF definition** | **PTH definition** | **KOG definition** | **GO definition** |
| --- | --- | --- | --- | --- | --- | --- | --- | --- |
| S3_5856578 | 5848089 | 5856696 | Manes.03G059600 | (PF) polyketide cyclase / dehydrase and lipid transport | PF10604 - polyketide cyclase / dehydrase and lipid transport | PTHR33789 - family not named |  |  |
|  |  |  | Manes.03G059700 |  |  |  |  |  |
|  |  |  | Manes.03G059800 | (PF) cotton fibre expressed protein | PF05553 - cotton fibre expressed protein | PTHR33265 - family not named; PTHR33265:SF2 - subfamily not named |  |  |
|  |  |  | Manes.03G059900 |  |  |  |  |  |
| S8_32339820 | 32304620 | 32318066 | Manes.08G161100 |  |  | PTHR33304 - family not named; PTHR33304:SF1 - subfamily not named |  | GO:0005515 - interacting selectively and non-covalently with any protein or protein complex (a complex of two or more proteins that may include other nonprotein molecules).; GO:0008270 - interacting selectively and non-covalently with zinc (zn) ions. |
|  | 32318199 | 32319086 | Manes.08G161200 |  |  |  |  |  |
|  | 32320251 | 32329335 | Manes.08G161300 |  |  | PTHR34048 - family not named; PTHR34048:SF3 - subfamily not named |  |  |
|  | 32324997 | 32325931 | Manes.08G161400 |  |  | PTHR24559:SF186 - subfamily not named; PTHR24559 - family not named |  |  |
|  | 32332142 | 32332837 | Manes.08G161500 |  |  |  |  | GO:0003676 - interacting selectively and non-covalently with any nucleic acid.; GO:0046872 - interacting selectively and non-covalently with any metal ion. |
|  | 32339376 | 32344790 | Manes.08G161600 | (PTH) 26s protease regulatory subunit; 26s protease regulatory subunit 6b | PF07728 - aaa domain (dynein-related subfamily) | PTHR23073 - 26s protease regulatory subunit; PTHR23073:SF8 - 26s protease regulatory subunit 6b |  | GO:0016787 - catalysis of the hydrolysis of various bonds, e.g. c-o, c-n, c-c, phosphoric anhydride bonds, etc. hydrolase is the systematic name for any enzyme of ec class 3.; GO:0005524 - interacting selectively and non-covalently with ATP, adenosine 5'-triphosphate, a universally important coenzyme and enzyme regulator.; GO:0005737 - all of the contents of a cell excluding the plasma membrane and nucleus, but including other subcellular structures.; GO:0030163 - the chemical reactions and pathways resulting in the breakdown of a protein by the destruction of the native, active configuration, with or without the hydrolysis of peptide bonds.; GO:0016887 - catalysis of the reaction: ATP + h2o = ADP + phosphate + 2 h+. may or may not be coupled to another reaction. |
|  | 32348829 | 32354394 | Manes.08G161700 |  |  |  |  | GO:0016491 - catalysis of an oxidation-reduction (redox) reaction, a reversible chemical reaction in which the oxidation state of an atom or atoms within a molecule is altered. one substrate acts as a hydrogen or electron donor and becomes oxidized, while the other acts as hydrogen or electron acceptor and becomes reduced.; GO:0055114 - a metabolic process that results in the removal or addition of one or more electrons to or from a substance, with or without the concomitant removal or addition of a proton or protons. |
|  | 32354818 | 32355489 | Manes.08G161800 | (PF) 3'-5' exonuclease | PF01612 - 3'-5' exonuclease | PTHR13620 - 3-5 exonuclease |  | GO:0006139 - any cellular metabolic process involving nucleobases, nucleosides, nucleotides and nucleic acids.; GO:0003676 - interacting selectively and non-covalently with any nucleic acid.; GO:0008408 - catalysis of the hydrolysis of ester linkages within nucleic acids by removing nucleotide residues from the 3' end. |
|  | 32356797 | 32357567 | Manes.08G161900 | (PF) 3'-5' exonuclease | PF01612 - 3'-5' exonuclease | PTHR13620 - 3-5 exonuclease |  | GO:0006139 - any cellular metabolic process involving nucleobases, nucleosides, nucleotides and nucleic acids.; GO:0003676 - interacting selectively and non-covalently with any nucleic acid.; GO:0008408 - catalysis of the hydrolysis of ester linkages within nucleic acids by removing nucleotide residues from the 3' end. |
|  | 32359053 | 32359391 | Manes.08G162000 |  |  |  |  |  |
|  | 32360530 | 32362230 | Manes.08G162100 | (PF) phosphatidylinositol 3- and 4-kinase | PF00454 - phosphatidylinositol 3- and 4-kinase | PTHR15245:SF23 - phosphatidylinositol 4-kinase gamma 1-related; PTHR15245 - symplekin-related | KOG2381 - phosphatidylinositol 4-kinase | GO:0016773 - catalysis of the transfer of a phosphorus-containing group from one compound (donor) to an alcohol group (acceptor). |
|  | 32377554 | 32383114 | Manes.08G162300 |  |  | PTHR33826 - family not named; PTHR33826:SF2 - hydroxyproline-rich glycoprotein family protein |  |  |
| S17_17327003 | 17299387 | 17301107 | Manes.17G040100 | (PF) organic solute transporter ostalpha | PF03619 - organic solute transporter ostalpha | PTHR23423:SF15 - subfamily not named; PTHR23423 - organic solute transporter-related | KOG2641 - predicted seven transmembrane receptor - rhodopsin family |  |
|  | 17301620 | 17301820 | Manes.17G040200 |  |  |  |  |  |
|  | 17302713 | 17311177 | Manes.17G040300 | (PF) galactose binding lectin domain; glycosyl hydrolases family 35 | PF02140 - galactose binding lectin domain; PF01301 - glycosyl hydrolases family 35 | PTHR23421 - beta-galactosidase related; PTHR23421:SF72 - subfamily not named |  | GO:0030246 - interacting selectively and non-covalently with any carbohydrate, which includes monosaccharides, oligosaccharides and polysaccharides as well as substances derived from monosaccharides by reduction of the carbonyl group (alditols), by oxidation of one or more hydroxy groups to afford the corresponding aldehydes, ketones, or carboxylic acids, or by replacement of one or more hydroxy group(s) by a hydrogen atom. cyclitols are generally not regarded as carbohydrates.; GO:0004553 - catalysis of the hydrolysis of any o-glycosyl bond.; GO:0005975 - the chemical reactions and pathways involving carbohydrates, any of a group of organic compounds based of the general formula cx(h2o)y. includes the formation of carbohydrate derivatives by the addition of a carbohydrate residue to another molecule. |
|  | 17313561 | 17317461 | Manes.17G040400 | (PF) s1/p1 nuclease | PF02265 - s1/p1 nuclease | PTHR33146 - family not named; PTHR33146:SF2 - endonuclease 2 |  | GO:0006308 - the cellular DNA metabolic process resulting in the breakdown of DNA, deoxyribonucleic acid, one of the two main types of nucleic acid, consisting of a long unbranched macromolecule formed from one or two strands of linked deoxyribonucleotides, the 3'-phosphate group of each constituent deoxyribonucleotide being joined in 3',5'-phosphodiester linkage to the 5'-hydroxyl group of the deoxyribose moiety of the next one.; GO:0004519 - catalysis of the hydrolysis of ester linkages within nucleic acids by creating internal breaks.; GO:0003676 - interacting selectively and non-covalently with any nucleic acid.; GO:0016788 - catalysis of the hydrolysis of any ester bond. |
|  | 17326226 | 17326372 | Manes.17G040500 |  |  | PTHR36615:SF2 - subfamily not named; PTHR36615 - family not named |  |  |
|  | 17326226 | 17326372 | Manes.17G040600 |  |  | PTHR36615 - family not named |  |  |
|  | 17333357 | 17339168 | Manes.17G040700 | (PF) scamp family | PF04144 - scamp family | PTHR10687:SF22 - secretory carrier-associated membrane protein 1-related; PTHR10687 - secretory carrier-associated membrane protein scamp | KOG3088 - secretory carrier membrane protein | GO:0016021 - the component of a membrane consisting of the gene products and protein complexes having at least some part of their peptide sequence embedded in the hydrophobic region of the membrane.; GO:0015031 - the directed movement of proteins into, out of or within a cell, or between cells, by means of some agent such as a transporter or pore. |
|  | 17346303 | 17354944 | Manes.17G040800 | (PF) protein of unknown function | PF05212 - protein of unknown function (duf707) | PTHR31210 - family not named; PTHR31210:SF11 - subfamily not named |  |  |
|  | 17357568 | 17360392 | Manes.17G040900 | (PF) myb-like DNA-binding domain; bromodomain | PF00249 - myb-like DNA-binding domain; PF00439 - bromodomain | PTHR15398 - bromodomain-containing protein 8; PTHR15398:SF5 - subfamily not named |  | GO:0005515 - interacting selectively and non-covalently with any protein or protein complex (a complex of two or more proteins that may include other nonprotein molecules).; GO:0003677 - any molecular function by which a gene product interacts selectively and non-covalently with DNA (deoxyribonucleic acid). |
|  | 17362752 | 17371871 | Manes.17G041000 | (PF) wd domain, g-beta repeat; con80 domain of katanin | PF00400 - wd domain, g-beta repeat; PF13925 - con80 domain of katanin | PTHR19845:SF5 - katanin p80 wd40 repeat-containing subunit B1 homolog; PTHR19845 - katanin p80 subunit | KOG0267 - microtubule severing protein katanin p80 subunit b (contains wd40 repeats) | GO:0005515 - interacting selectively and non-covalently with any protein or protein complex (a complex of two or more proteins that may include other nonprotein molecules). |
| S18_3408138 | 3397926 | 3400465 | Manes.18G039200 | (PF) Peptidase inhibitor I9; PTHR10795 - proprotein convertase subtilisin/kexin | PF00082 - Subtilase family; PF05922 - Peptidase inhibitor I9 | PTHR10795 - proprotein convertase subtilisin/kexin; PTHR10795:SF394 - subfamily not named | KOG1153 - Subtilisin-related protease/Vacuolar protease B | GO:0006508 - The hydrolysis of proteins into smaller polypeptides and/or amino acids by cleavage of their peptide bonds.; GO:0004252 - Catalysis of the hydrolysis of internal, alpha-peptide bonds in a polypeptide chain by a catalytic mechanism that involves a catalytic triad consisting of a serine nucleophile that is activated by a proton relay involving an acidic residue (e.g. aspartate or glutamate) and a basic residue (usually histidine). |
| S18_2907312 | 2884707 | 2886777 | Manes.18G032300 | (PF) 3-beta hydroxysteroid dehydrogenase/isomerase family | PF01073 - 3-beta hydroxysteroid dehydrogenase/isomerase family | PTHR10366 - nad dependent epimerase/dehydratase; PTHR10366:SF400 - subfamily not named | KOG1502 - flavonol reductase/cinnamoyl-coa reductase | GO:0006694 - the chemical reactions and pathways resulting in the formation of steroids, compounds with a 1,2,cyclopentanoperhydrophenanthrene nucleus; includes de novo formation and steroid interconversion by modification.; GO:0016616 - catalysis of an oxidation-reduction (redox) reaction in which a ch-oh group acts as a hydrogen or electron donor and reduces nad+ or nADP.; GO:0055114 - a metabolic process that results in the removal or addition of one or more electrons to or from a substance, with or without the concomitant removal or addition of a proton or protons.; GO:0003854 - catalysis of the reaction: a 3-beta-hydroxy-delta(5)-steroid + nad+ = a 3-oxo-delta(5)-steroid + nadh + h(+). |
|  | 2887205 | 2890928 | Manes.18G032400 |  |  |  |  |  |
|  | 2894179 | 2899813 | Manes.18G032500 |  |  | PTHR33344:SF1 - subfamily not named; PTHR33344 - family not named |  |  |
|  | 2900678 | 2903482 | Manes.18G032600 |  |  |  |  | GO:0005515 - interacting selectively and non-covalently with any protein or protein complex (a complex of two or more proteins that may include other nonprotein molecules). |
|  | 2904195 | 2904962 | Manes.18G032700 |  |  | PTHR36324 - family not named; PTHR36324:SF1 - subfamily not named |  |  |
|  | 2905268 | 2907503 | Manes.18G032800 | (PF) domain of unknown function | PF14290 - domain of unknown function (duf4370) | PTHR36139:SF1 - subfamily not named; PTHR36139 - family not named |  |  |
|  | 908201 | 2909378 | Manes.18G032900 | (PF) carboxylesterase family | PF00135 - carboxylesterase family | PTHR23024 - member of 'gdxg' family of lipolytic enzymes; PTHR23024:SF235 - subfamily not named | KOG1515 - arylacetamide deacetylase |  |
|  | 2909496 | 2910783 | Manes.18G033000 | (PF) alpha/beta hydrolase family | PF12695 - alpha/beta hydrolase family | PTHR23024 - member of 'gdxg' family of lipolytic enzymes; PTHR23024:SF131 - carboxylesterase 2-related | KOG1515 - arylacetamide deacetylase |  |
|  | 2913443 | 2916514 | Manes.18G033100 | (PF) maintenance of mitochondrial structure and function; prokaryotic homologs of the jab domain | PF13012 - maintenance of mitochondrial structure and function; PF14464 - prokaryotic homologs of the jab domain | PTHR10410:SF5 - 26s proteasome non-ATPase regulatory subunit 14; PTHR10410 - eukaryotic translation initiation factor 3 -related | KOG1555 - 26s proteasome regulatory complex, subunit rpn11 | GO:0005515 - interacting selectively and non-covalently with any protein or protein complex (a complex of two or more proteins that may include other nonprotein molecules). |
|  | 2919559 | 2921611 | Manes.18G033200 | (PF) Patatin-like phospholipase | PF01734 - Patatin-like phospholipase | PTHR32176 - family not named | KOG0513 - ca2+-independent phospholipase a2 | GO:0006629 - the chemical reactions and pathways involving lipids, compounds soluble in an organic solvent but not, or sparingly, in an aqueous solvent. includes fatty acids; neutral fats, other fatty-acid esters, and soaps; long-chain (fatty) alcohols and waxes; sphingoids and other long-chain bases; glycolipids, phospholipids and sphingolipids; and carotenes, polyprenols, sterols, terpenes and other isoprenoids.; GO:0008152 - the chemical reactions and pathways, including anabolism and catabolism, by which living organisms transform chemical substances. metabolic processes typically transform small molecules, but also include macromolecular processes such as DNA repair and replication, and protein synthesis and degradation. |
|  | 2928220 | 2929151 | Manes.18G033300 | (PF) Patatin-like phospholipase | PF01734 - Patatin-like phospholipase | PTHR32176 - family not named |  | GO:0006629 - the chemical reactions and pathways involving lipids, compounds soluble in an organic solvent but not, or sparingly, in an aqueous solvent. includes fatty acids; neutral fats, other fatty-acid esters, and soaps; long-chain (fatty) alcohols and waxes; sphingoids and other long-chain bases; glycolipids, phospholipids and sphingolipids; and carotenes, polyprenols, sterols, terpenes and other isoprenoids.; GO:0008152 - the chemical reactions and pathways, including anabolism and catabolism, by which living organisms transform chemical substances. metabolic processes typically transform small molecules, but also include macromolecular processes such as DNA repair and replication, and protein synthesis and degradation. |
|  | 2933320 | 2935156 | Manes.18G033400 | (PF) Patatin-like phospholipase | PF01734 - Patatin-like phospholipase | PTHR32176 - family not named | KOG0513 - ca2+-independent phospholipase a2 | GO:0006629 - the chemical reactions and pathways involving lipids, compounds soluble in an organic solvent but not, or sparingly, in an aqueous solvent. includes fatty acids; neutral fats, other fatty-acid esters, and soaps; long-chain (fatty) alcohols and waxes; sphingoids and other long-chain bases; glycolipids, phospholipids and sphingolipids; and carotenes, polyprenols, sterols, terpenes and other isoprenoids.; GO:0008152 - the chemical reactions and pathways, including anabolism and catabolism, by which living organisms transform chemical substances. metabolic processes typically transform small molecules, but also include macromolecular processes such as DNA repair and replication, and protein synthesis and degradation. |
|  | 2939118 | 2940726 | Manes.18G033500 | (PF) heme oxygenase | PF01126 - heme oxygenase | PTHR10720:SF3 - subfamily not named; PTHR10720 - heme oxygenase |  | GO:0004392 - catalysis of the reaction: heme + 3 donor-h2 + 3 o2 = biliverdin + fe2+ + co + 3 acceptor + 3 h2o.; GO:0006788 - the chemical reactions and pathways resulting in the loss of electrons from one or more atoms in heme.; GO:0055114 - a metabolic process that results in the removal or addition of one or more electrons to or from a substance, with or without the concomitant removal or addition of a proton or protons. |
|  | 2942262 | 2942378 | Manes.18G033600 |  |  |  |  |  |
|  | 2943620 | 2945874 | Manes.18G033700 |  |  | PTHR32175 - family not named; PTHR32175:SF7 - subfamily not named |  |  |
| S18_3081635 | 3046841 | 3051621 | Manes.18G035300 | (PF) transmembrane amino acid transporter protein | PF01490 - transmembrane amino acid transporter protein | PTHR22950 - amino acid transporter; PTHR22950:SF281 - lysine histidine transporter-like 8 | KOG1303 - amino acid transporters |  |
|  | 3053441 | 3054157 | Manes.18G035400 | (PF) Dof domain, zinc finger | PF02701 - Dof domain, zinc finger | PTHR31992 - family not named; PTHR31992:SF12 - dof zinc finger protein dof3.4 |  | GO:0003677 - any molecular function by which a gene product interacts selectively and non-covalently with DNA (deoxyribonucleic acid).; GO:0006355 - any process that modulates the frequency, rate or extent of cellular DNA-templated transcription. |
|  | 3081656 | 3091683 | Manes.18G035500 | (PF) protein phosphatase 2c | PF00481 - protein phosphatase 2c | PTHR13832:SF25 - protein phosphatase 2c 12-related; PTHR13832 - protein phosphatase 2c | KOG0698 - serine/threonine protein phosphatase | GO:0004722 - catalysis of the reaction: protein serine phosphate + h2o = protein serine + phosphate, and protein threonine phosphate + h2o = protein threonine + phosphate.; GO:0003824 - catalysis of a biochemical reaction at physiological temperatures. in biologically catalyzed reactions, the reactants are known as substrates, and the catalysts are naturally occurring macromolecular substances known as enzymes. enzymes possess specific binding sites for substrates, and are usually composed wholly or largely of protein, but rna that has catalytic activity (ribozyme) is often also regarded as enzymatic.; GO:0006470 - the process of removing one or more phosphoric residues from a protein. |
|  | 3091699 | 3095692 | Manes.18G035600 | (PTH) BRI1 kinase inhibitor 1 |  | PTHR33312:SF8 - BRI1 kinase inhibitor 1; PTHR33312 - family not named |  |  |
|  | 3097779 | 3106261 | Manes.18G035700 | (PF) lipin, n-terminal conserved region; lns2 (lipin/ned1/smp2) | PF04571 - lipin, n-terminal conserved region; PF08235 - lns2 (lipin/ned1/smp2) | PTHR12181:SF12 - protein lpin-1; PTHR12181 - lipin | KOG2116 - protein involved in plasmid maintenance/nuclear protein involved in lipid metabolism |  |
|  | 3106385 | 3106675 | Manes.18G035800 | (PTH) EF-hand calcium-binding domain containing protein | PF13202 - ef hand | PTHR10891 - EF-hand calcium-binding domain containing protein; PTHR10891:SF575 - subfamily not named |  | GO:0005509 - interacting selectively and non-covalently with calcium ions (ca2+). |
|  | 3107584 | 3107874 | Manes.18G035900 | (PTH) EF-hand calcium-binding domain containing protein |  |  |  |  |
|  | 3108459 | 3109073 | Manes.18G036000 | (PTH) EF-hand calcium-binding domain containing protein | PF13202 - ef hand | PTHR10891 - EF-hand calcium-binding domain containing protein; PTHR10891:SF597 - subfamily not named |  | GO:0005509 - interacting selectively and non-covalently with calcium ions (ca2+). |
|  | 3111508 | 3112165 | Manes.18G036100 | (PTH) EF-hand calcium-binding domain containing protein; calcium-binding protein cml38-related | PF13499 - ef-hand domain pair | PTHR10891 - EF-hand calcium-binding domain containing protein; PTHR10891:SF592 - calcium-binding protein cml38-related |  | GO:0005509 - interacting selectively and non-covalently with calcium ions (ca2+). |
|  | 3114611 | 3115118 | Manes.18G036200 | (PTH) EF-hand calcium-binding domain containing protein; calcium-binding protein cml38-related | PF13202 - ef hand | PTHR10891 - EF-hand calcium-binding domain containing protein; PTHR10891:SF592 - calcium-binding protein cml38-related |  | GO:0005509 - interacting selectively and non-covalently with calcium ions (ca2+). |
|  | 3117265 | 3118990 | Manes.18G036300 | (PTH) EF-hand calcium-binding domain containing protein; calcium-binding protein cml38-related | PF13202 - ef hand | PTHR10891 - EF-hand calcium-binding domain containing protein; PTHR10891:SF592 - calcium-binding protein cml38-related |  | GO:0005509 - interacting selectively and non-covalently with calcium ions (ca2+). |
| S18_3399799 | 3406921 | 3409596 | Manes.18G039300 | (PF) peptidase inhibitor i9; PTHR10795 - proprotein convertase subtilisin/kexin | PF00082 - subtilase family; PF05922 - peptidase inhibitor i9 | PTHR10795 - proprotein convertase subtilisin/kexin; PTHR10795:SF373 - subfamily not named |  | GO:0006508 - the hydrolysis of proteins into smaller polypeptides and/or amino acids by cleavage of their peptide bonds.; GO:0004252 - catalysis of the hydrolysis of internal, alpha-peptide bonds in a polypeptide chain by a catalytic mechanism that involves a catalytic triad consisting of a serine nucleophile that is activated by a proton relay involving an acidic residue (e.g. aspartate or glutamate) and a basic residue (usually histidine). |
| S18_3399801 |  |  | Manes.18G039400 | PTHR27001:sf90 - protein kinase family protein | PF00069 - protein kinase domain | PTHR27001 - family not named; PTHR27001:sf90 - protein kinase family protein | KOG1187 - serine/threonine protein kinase | GO:0006468 - the process of introducing a phosphate group on to a protein.; GO:0004713 - catalysis of the reaction: ATP + a protein tyrosine = ADP + protein tyrosine phosphate.; GO:0004672 - catalysis of the phosphorylation of an amino acid residue in a protein, usually according to the reaction: a protein + ATP = a phosphoprotein + ADP.; GO:0005524 - interacting selectively and non-covalently with ATP, adenosine 5'-triphosphate, a universally important coenzyme and enzyme regulator. |
|  |  |  | Manes.18G039500 | (PF) helix-loop-helix DNA-binding domain | PF00010 - helix-loop-helix DNA-binding domain | PTHR11514 - myc; PTHR11514:SF57 - subfamily not named |  | GO:0016597 - interacting selectively and non-covalently with an amino acid, organic acids containing one or more amino substituents.; GO:0046983 - the formation of a protein dimer, a macromolecular structure consists of two noncovalently associated identical or nonidentical subunits.; GO:0008152 - the chemical reactions and pathways, including anabolism and catabolism, by which living organisms transform chemical substances. metabolic processes typically transform small molecules, but also include macromolecular processes such as DNA repair and replication, and protein synthesis and degradation. |
|  | 3421767 | 3424121 | Manes.18G039600 | (PF) helix-loop-helix DNA-binding domain | PF00010 - helix-loop-helix DNA-binding domain | PTHR12565 - sterol regulatory element-binding protein; PTHR12565:sf92 - transcription factor spatula |  | GO:0046983 - the formation of a protein dimer, a macromolecular structure consists of two noncovalently associated identical or nonidentical subunits. |
|  |  |  | Manes.18G039700 | (PTH) ring finger containing protein; ring finger domain-containing | PF13639 - ring finger domain | PTHR22937 - ring finger containing protein; PTHR14155 - ring finger domain-containing; PTHR22937:SF55 - subfamily not named; PTHR14155:SF72 - subfamily not named |  | GO:0005515 - interacting selectively and non-covalently with any protein or protein complex (a complex of two or more proteins that may include other nonprotein molecules).; GO:0008270 - interacting selectively and non-covalently with zinc (zn) ions. |
|  | 3431993 | 3432696 | Manes.18G039800 |  |  |  |  |  |
|  |  |  | Manes.18G039900 | (PTH) ring finger domain-containing | PF13639 - ring finger domain | PTHR14155 - ring finger domain-containing; PTHR14155:SF72 - subfamily not named |  | GO:0005515 - interacting selectively and non-covalently with any protein or protein complex (a complex of two or more proteins that may include other nonprotein molecules).; GO:0008270 - interacting selectively and non-covalently with zinc (zn) ions. |
| S18_3407893 | 3373459 | 3375082 | Manes.18G039100 | PTHR15451:SF19 - ergosterol biosynthetic protein 28-related | PF03694 - Erg28 like protein | PTHR15451:SF19 - ergosterol biosynthetic protein 28-related; PTHR15451 - family not named |  | GO:0016021 - The component of a membrane consisting of the gene products and protein complexes having at least some part of their peptide sequence embedded in the hydrophobic region of the membrane. |
| S18_3567791 |  |  | Manes.18G040700 |  |  | PTHR35513 - family not named |  |  |
| S18_3567816 |  |  | Manes.18G040800 | (PF) protein tyrosine kinase; leucine rich repeat | PF07714 - protein tyrosine kinase; PF13855 - leucine rich repeat | PTHR27008 - family not named; PTHR27008:SF39 - subfamily not named | KOG1187 - serine/threonine protein kinase | GO:0006468 - the process of introducing a phosphate group on to a protein.; GO:0004713 - catalysis of the reaction: ATP + a protein tyrosine = ADP + protein tyrosine phosphate.; GO:0004672 - catalysis of the phosphorylation of an amino acid residue in a protein, usually according to the reaction: a protein + ATP = a phosphoprotein + ADP.; GO:0005515 - interacting selectively and non-covalently with any protein or protein complex (a complex of two or more proteins that may include other nonprotein molecules).; GO:0005524 - interacting selectively and non-covalently with ATP, adenosine 5'-triphosphate, a universally important coenzyme and enzyme regulator. |
|  |  |  | Manes.18G040900 |  |  | PTHR27000:SF167 - subfamily not named; PTHR27000 - family not named |  |  |
|  |  |  | Manes.18G041000 | (PTH) WRKY transcription factor 7-related; (PF) plant zinc cluster domain | PF03106 - WRKY DNA -binding domain; PF10533 - plant zinc cluster domain | PTHR31282:SF27 - WRKY transcription factor 7-related; PTHR31282 - family not named |  | GO:0043565 - interacting selectively and non-covalently with DNA of a specific nucleotide composition, e.g. gc-rich DNA binding, or with a specific sequence motif or type of DNA e.g. promotor binding or rDNA binding.; GO:0003700 - interacting selectively and non-covalently with a specific DNA sequence in order to modulate transcription. the transcription factor may or may not also interact selectively with a protein or macromolecular complex.; GO:0006355 - any process that modulates the frequency, rate or extent of cellular DNA-templated transcription. |
|  |  |  | Manes.18G041100 | (PTH) cysteine protease family c1-related; cysteine protease component of protease-inhibitor complex-related | PF00396 - granulin; PF00112 - papain family cysteine protease; PF08246 - cathepsin propeptide inhibitor domain (i29) | PTHR12411 - cysteine protease family c1-related; PTHR12411:SF344 - cysteine protease component of protease-inhibitor complex-related | KOG1543 - cysteine proteinase cathepsin l | GO:0006508 - the hydrolysis of proteins into smaller polypeptides and/or amino acids by cleavage of their peptide bonds.; GO:0008234 - catalysis of the hydrolysis of peptide bonds in a polypeptide chain by a mechanism in which the sulfhydryl group of a cysteine residue at the active center acts as a nucleophile. |
|  |  |  | Manes.18G041200 | (PTH) mannose-p-dolichol utilization defect 1 protein; mannose-p-dolichol utilization defect 1 lec35 -related | PF04193 - pq loop repeat | PTHR12226:SF2 - mannose-p-dolichol utilization defect 1 protein; PTHR12226 - mannose-p-dolichol utilization defect 1 lec35 -related | KOG3211 - predicted endoplasmic reticulum membrane protein lec35/mpdu1 involved in monosaccharide-p-dolichol utilization |  |
|  | 3567333 | 3568084 | Manes.18G041300 |  |  | PTHR33033 - family not named |  | GO:0003676 - interacting selectively and non-covalently with any nucleic acid. |
|  |  |  | Manes.18G041400 | (PTH) f-box protein skip2 | PF00646 - F-box domain | PTHR24006 - family not named; PTHR24006:SF481 - f-box protein skip2 | KOG1947 - leucine rich repeat proteins, some proteins contain f-box | GO:0005515 - interacting selectively and non-covalently with any protein or protein complex (a complex of two or more proteins that may include other nonprotein molecules). |
|  |  |  | Manes.18G041600 | (PTH) cycline | PF02984 - cyclin, c-terminal domain; PF00134 - cyclin, n-terminal domain | PTHR10177 - cycline; PTHR10177:SF242 - subfamily not named | KOG0656 - g1/s-specific cyclin d | GO:0005634 - a membrane-bounded organelle of eukaryotic cells in which chromosomes are housed and replicated. in most cells, the nucleus contains all of the cell's chromosomes except the organellar chromosomes, and is the site of rna synthesis and processing. in some species, or in specialized cell types, rna metabolism or DNA replication may be absent. |
| S18_13522313 | 14132468 | 14142820 | Manes.18G124700 |  |  | PTHR31016 - uncharacterized; PTHR31016:SF3 - subfamily not named |  |  |
|  | 14137005 | 14146619 | Manes.18G124800 | (PF) ring finger domain | PF13639 - ring finger domain | PTHR15860 - uncharacterized ring finger-containing protein; PTHR15860:SF4 - subfamily not named | KOG4172 - predicted e3 ubiquitin ligase | GO:0005515 - interacting selectively and non-covalently with any protein or protein complex (a complex of two or more proteins that may include other nonprotein molecules).; GO:0008270 - interacting selectively and non-covalently with zinc (zn) ions. |
|  | 14155813 | 14159274 | Manes.18G124900 |  |  |  |  |  |
