# Supplementary material for: Genome-wide association study of cassava starch paste properties
Source: PLoS One. 2022 Jan 21;17(1):e0262888. doi: 10.1371/journal.pone.0262888 (PMC8782291; doi:10.1371/journal.pone.0262888)
Supplement: Attachment — Submitted filename: Reviewer Comments - PLOSE ONE manuscript.docx [file pone.0262888.s012.docx]

**Reviewer Comments**

**Manuscript Title: Genome-wide association study of cassava starch paste properties**

**Title: Adequate**

**General comment: This manuscript utilized a diverse set of cassava population comprising landraces and improved cultivars from different countries for GWAS in the dissection of the genomic regions underlying cassava pasting properties based on different methods: MLM, MLMM and FarmCPU. To the best of the Authors’ knowledge, this is the first of such studies employing GWAS for mapping of such pasting property - related traits [pasting temperature, peak viscosity, hot-paste viscosity, cool-paste viscosity, final viscosity, breakdown, and setback] .in cassava. The present study is an important contribution in the field; the set of experiments and data analyses were well conducted; and the manuscript properly organized. I found this manuscript to be of sufficient merit and recommended for publication in PLOSE One after minor corrections.**

**I have made minor corrections and raised some comments and questions in the marked manuscript with tracked-changes which should be addressed by the authors.**

**Abstract: Ok.**

**Introduction: Ok. Clearly written and sufficient background of study provided**

**Materials and Methods: Adequate and well described**

**Results: Adequate. However, On S2 Table, Total and mean should be included at the bottom. This will aid readers to quickly know the Total size (Mb) of the 18 Chromosomes and also the total number of Markers (followed by the mean value per chromosome as you have already indicated). I could not find Table S1 among the supporting documents or in the main text (pdf).**

**Discussion: The results obtained were well discussed. Minor points were raised in a few aspects**

**Final Decision: The manuscript is of sufficient merit for Publication in PLOSE ONE. All the minor corrections and the additional comment should be taken into consideration.**

**I therefore recommended acceptance for publication with minor revision.**

**Prof. Benjamin E. Ubi**
